# Dietary supplementation with L-carnitine elevates intracellular carnitine levels and affects gene expression of SLC25A20 and COX4I1, as well as non-mitochondrial respiration of bovine blood cells during systemic immune challenge

**DOI:** 10.3389/fimmu.2025.1583351

**Published:** 2025-07-11

**Authors:** Leonie Seemann, Susanne Kersten, Susanne Bühler, Fabian Billenkamp, Ulrich Meyer, Christian Visscher, Korinna Huber, Erika Most, Klaus Eder, Sven Dänicke, Jana Frahm

**Affiliations:** ^1^ Institute of Animal Nutrition, Friedrich-Loeffler-Institut, Federal Research Institute for Animal Health, Braunschweig, Germany; ^2^ Institute of Animal Nutrition, University of Veterinary Medicine Hannover, Foundation, Hannover, Germany; ^3^ Department of Functional Anatomy of Livestock, Institute of Animal Science, University of Hohenheim, Stuttgart, Germany; ^4^ Institute of Animal Nutrition and Nutrition Physiology, Justus-Liebig-University, Gießen, Germany

**Keywords:** L-carnitine, dairy cow, mid-lactation, lipopolysaccharide, PBMC

## Abstract

L-carnitine plays a key role in shuttling free fatty acids from the cytosol into the mitochondrial matrix. Fatty acids, among other substrates, are utilized by immune cells as an energy source. Therefore, L-carnitine, which is authorized as a feed additive in cattle, may influence the metabolism of peripheral blood mononuclear cells (PBMC) during an immune challenge. To test this hypothesis, a feeding trial was conducted with 53 German Holstein cows, comprising a control group (CON, n = 26) and an L-carnitine supplemented group (CAR, n = 27, 25 g rumen-protected L-carnitine/cow/d). On day 111 after calving, all cows were intravenously injected with lipopolysaccharides (LPS, 0.5 µg/kg body weight as bolus injection, *E. coli*) to induce a systemic immune challenge. Blood samples were collected on day 143 *ante injectionem (ai)*, day 11 *ai*, 24 hours *post injectionem* (*pi*), and day 14 *pi* and PBMC were isolated. The used methods included high-performance liquid chromatography coupled with mass spectrometry, Alamar Blue assay, real-time qPCR, and the Mito Stress Test of the Seahorse Analyzer (Agilent, Santa Clara, California, USA). L-carnitine supplementation significantly increased intracellular concentrations of carnitine and its precursor γ-butyrobetaine in PBMC of dairy cows. The gene expression of carnitine-acylcarnitine translocase (SLC25A20) in PBMC remained stable in CAR, whereas it was upregulated in CON during the LPS challenge, suggesting an adaptation to increased energy demands in CON. A contrasting pattern was detected for the gene expression of cytochrome c oxidase subunit 4I1 (COX4I1), with stable levels in CON and a downregulation in CAR due to LPS injection. However, most of the investigated genes were unaffected by L-carnitine supplementation, and responded significantly to LPS injection. The same applied for PBMC mitochondrial functionality and metabolic activity as assessed by ex vivo approaches, whereas non-mitochondrial respiration rate was significantly affected by L-carnitine supplementation over time. In conclusion, dietary L-carnitine supplementation of 25 g per cow per day led to a balanced distribution of carnitine and γ-butyrobetaine between bovine blood cells and plasma, but there were only minor effects on gene level and cellular respiration.

## 1 Introduction

Maintaining and improving the health of dairy cows is an important issue for farmers, as it directly impacts the productivity and longevity of their livestock (1). In order to support this objective, a variety of feed additives are supposed to enhance physiological functions (2). L-carnitine is one of these supplements and plays a pivotal role in cellular energy metabolism, as it is essential for the transport of long-chain fatty acids (LCFA) from the cytosol into the mitochondrial matrix, where LCFA are oxidized (3). In this mechanism, L-carnitine is acylated with activated LCFA by carnitine-palmitoyl transferase 1, which is located on the outer mitochondrial membrane (4). Critically, these molecules can only cross the inner mitochondrial membrane with the carnitine-acylcarnitine translocase, which exchanges acylcarnitine for carnitine (4). In the mitochondrial matrix, the acyl group is removed by carnitine-palmitoyl transferase 2, allowing L-carnitine to exit the matrix via the translocase (4).

L-carnitine can be synthesized endogenously from the amino acids lysine and methionine (5) and can also be absorbed by dairy cows with the feed, as evidenced by elevated blood carnitine levels in supplemented cows (6). In humans, L-carnitine has been demonstrated to support recovery from exercise (7) and in aged rats it has been shown to modulate macrophage function (8). It is assumed that L-carnitine metabolism is similar in all mammals (9), and therefore L-carnitine supplementation may also have benefits for dairy cows, particularly in supporting the immune system to cope with energy demanding situations, as e. g. the acute phase reaction induced by recognition of pathogen-associated molecular patterns (PAMPs).

The systemic administration of lipopolysaccharides (LPS), which are PAMPs of the membrane of gram-negative bacteria, can experimentally induce an acute phase reaction, resulting in a high energy demand for the immune system (10). Intravenous LPS injection has been shown to activate the innate immune system of dairy cows, for instance, cytokine production, leukocyte count, and leukocyte functionality (11, 12).

This study focuses on peripheral blood mononuclear cells (PBMC), a diverse population of immune cells primarily consisting of different lymphocytes and monocytes (13), which showed substantial changes during the immune challenge (11). As part of the present study, absolute counts of monocytes and lymphocytes decreased sharply immediately after intravenous LPS injection in dairy cows, reaching baseline values again within 14 d after the immune challenge (11). Additionally, the proportion of phagocytosing PBMC, as well as their phagocytic capacity, increased after the LPS injection (11).

In the field of immunological research, PBMC are a valuable cell type *in vivo* as well as *ex vivo*, to examine cellular metabolism under different conditions (13). As they are a mixed cell population, they may use different types of energy sources, like glycolysis, glutaminolysis, and also fatty acid oxidation (14). Therefore, these cells could benefit from L-carnitine supplementation during an immune challenge.

The present study aimed to gain deeper insights into the metabolism of PBMC under the conditions of L-carnitine oversupply and increased energy demand. It was hypothesized that the metabolism of PBMC would be supported by dietary L-carnitine supplementation with specific modulations at the cellular level and gene expression that improve immune functionality during an energy-demanding challenge.

## 2 Materials and methods

### 2.1 Animal experiment and sampling

The study was conducted at the experimental station of the Institute of Animal Nutrition, Friedrich-Loeffler-Institut (FLI), located in Braunschweig, Germany, in full compliance with the German Animal Welfare Act and approved by the Lower Saxony State Office for Consumer Protection and Food Safety (LAVES, Oldenburg, Germany) (AZ33.19-42502-04-16/2378).

This trial is part of a larger experiment that lasted from d 42 *ante partum (ap)* to d 128 *post partum (pp)*. Full details of the experimental design and feeding regimen have been previously published by Meyer et al. (6, 15), Kononov et al. (16, 17), and Seemann et al. (11). In summary, the study included 53 pluriparous German Holstein cows assigned to an L-carnitine supplemented group (CAR; n = 27) and a control group (CON; n = 26). These groups were balanced for body condition score (BCS, 3.33 ± 0.51), body weight (BW, 705 kg ± 75 kg), and number of lactation (2.6 ± 0.8). The cows were fed a partial mixed ration according to the guidelines of the Society of Nutritional Physiology (GfE, 2001) for nutrient and energy supply, consisting of 50% roughage (70% maize silage and 30% grass silage) administered in feed-weigh troughs (Roughage Intake Control, System Insentec B.V., Marknesse, The Netherlands) and 50% concentrate feed, administered in electronic feeding stations (Insentec B.V., Marknesse, The Netherlands). CAR received a daily supplement of 125 g per cow of a rumen-protected L-carnitine product (Carneon 20 Rumin-Pro, Kaesler Nutrition GmbH, Cuxhaven, Germany) with the concentrate feed, resulting in 25 g of L-carnitine per cow and day, while CON received a supplement to equalize the fat content (BergaFat F-100 HP, Berg + Schmidt GmbH & Co. KG, Hamburg, Germany). The cows had unlimited access to water. The present study focused on the timeframe between d 100 *pp* and d 128 *pp*. On d 111 *pp*, all cows were administered 0.5 µg/kg BW LPS (*E. coli*, Serotype O111:B4, Sigma Aldrich, L2630, St. Louis, Missouri, USA) by needle puncture into a jugular vein. Two cows were excluded from the experiment due to unphysiologically low rectal temperatures before the LPS challenge. In addition, one cow died 24 h *post injectionem (pi)* due to an acute shock, probably due to unrecognized inflammation. Thus, the study was completed with 50 cows (n_CON_ = 24, n_CAR_ = 26).

Blood was collected by needle puncture into lithium heparin vacutainers^®^ (Becton Dickinson GmbH, Heidelberg, Germany) and EDTA tubes (Sarstedt AG & Co. KG, Nümbrecht, Germany) at the following time points: 143 d *ante injectionem (ai)* (= d 42 *ap*, before L-carnitine supplementation), 11 *d ai* (= d 100 *pp*), 24 h *pi* (= d 112 *pp*) and 14 d *pi* (= d 126 *pp*).

### 2.2 Isolation of PBMC

Bovine PBMC were isolated from heparinized blood by density gradient centrifugation immediately after sampling. The blood samples were diluted 1:2 in phosphate-buffered saline (PBS), layered onto a separation solution (Biocoll 1.077 g/ml, Biochrom AG, Berlin, Germany), and centrifuged (800 x g, 20 min, 20°C, Multifuge X3 F2, Thermo Fisher Scientific, Dreieich, Germany). The resulting buffy coat was collected, resuspended in PBS, and centrifuged again (200 x g, 8 min, 20°C, Universal 320, Hettich, Tuttlingen, Germany). Erythrocyte lysis was then carried out using NaCl (8.8%) and distilled H_2_O, followed by another centrifugation step. PBMC pellets were resuspended in Roswell Park Memorial Institute (RPMI)-medium without additives. After a further centrifugation (200 x g, 8 min, 20°C, Universal 320, Andreas Hettich GmbH, Tuttlingen, Germany), PBMC were resuspended in RPMI medium supplemented with fetal bovine serum, HEPES buffer, L-glutamine, and β-mercaptoethanol (all purchased from Biochrom AG, Berlin, Germany). The number of dead cells was determined by staining with trypan blue, and cells were counted using a Neubauer hemocytometer. After initial analyses, the remaining cells were centrifuged and stored as pellets at -80°C for further investigations.

### 2.3 Intracellular carnitine and derivatives

The concentrations of carnitine, γ-butyrobetaine (γBB), trimethyllysine (TML) and acetlycarnitine were determined in PBMC and erythrocyte lysate by tandem mass spectrometry according to Hirche et al. (18).

#### 2.3.1 PBMC

In brief, the stored PBMC pellets were thawed, buffered with Tris base, and lysed by sonication (15 min, 30°C). For the extraction, methanol and internal standards were added, followed by incubation for 15 min at 30°C on a thermal shaker (650 rpm, TS1 ThermoShaker, Biometra, Göttingen, Germany), centrifugation (21,000 x g, 10 min, 4°C, Heraeus Fresco 21 Centrifuge, Thermo Scientific, Schwerte, Germany), evaporation, and centrifugation with methanol again. The resulting supernatant was analyzed using high-performance liquid chromatography (HPLC, VWR Hitachi, Radnor, Pennsylvania, USA) coupled with mass spectrometry (3200 Q Trap, AB Sciex, Framingham, Massachusetts, USA) to quantify the target compounds. Furthermore, the protein content was determined in lysed PBMC to generate a normalization factor, using a bicinchoninic acid assay (BC Assay, Uptima, Interchim, Montluçon, France).

#### 2.3.2 Erythrocyte lysate

EDTA blood mixed with cold distilled water was centrifuged (10,000 x g, 10 min, 4°C, Rovall RC6+, Thermo Fisher Scientific, Dreieich, Germany) and the top layer forming the erythrocyte lysate (erylysate) was stored at -80°C before analysis. After thawing, the samples were diluted 1:2 with chromatography water followed by sonication (25 min, 20°C, SONOREX RK 100, Bandelin, Berlin, Germany) and centrifugation (21,000 x g, 10 min, 4°C, Heraeus Fresco 21 Centrifuge, Thermo Scientific, Schwerte, Germany). Thereafter, methanol and internal standards were added and incubated at 5°C followed by centrifugation again. Carnitine and its derivatives were immediately determined in the supernatant using high-performance liquid chromatography (HPLC, VWR Hitachi, Radnor, Pennsylvania, USA) coupled with mass spectrometry (3200 Q Trap, AB Sciex, Framingham, Massachusetts, USA). The hemoglobin concentration served as normalization factor and was measured immediately after sampling in EDTA whole blood using the automated analyzer Celltac-α (MEK 6450, Nihon Kohden, Qinlab Diagnostik, Weichs, Germany).

### 2.4 ConA-stimulated proliferation of PBMC

The metabolic activity of PBMC was investigated using the Alamar Blue (AB) assay. The method is based on metabolically active cells reducing non-fluorescent resazurin to fluorescent resorufin. Immediately after isolation, PBMC were seeded on a 96-well microplate at a density of 10^5^ cells per well and incubated for 69.5 h at 37°C and 5% CO_2_ with or without stimulation of concanavalin A (ConA, 2.5 µg/ml; Sigma-Aldrich, Steinheim, Germany). Subsequently, Alamar Blue (AbD Serotec, Oxford, UK) was added at a ratio of 1:10, and the cells were incubated for an additional 2.5 hours. The fluorescence of resorufin was then measured at an excitation wavelength of 540 nm and an emission wavelength of 590 nm using a photometer (Tecan Infinite^®^ M200, Tecan Group Ltd., Männedorf, Switzerland). The results from five replicates were calculated and expressed as stimulation index (SI).

### 2.5 Gene expression of PBMC

#### 2.5.1 RNA extraction

PBMC resuspended in PBS were centrifuged (400 x g, 12 min, 4°C, Varifuge 3.0, Heraeus, Hanau, Germany) immediately after density gradient centrifugation. Erythrocytes were lysed twice by adding DEPC-treated H_2_O and NaCl (8.8%), followed by centrifugation (450 x g, 6 min, 4°C, Universal 320 R, Hettich, Tuttlingen, Germany). The resulting PBMC pellets were resuspended in lysis buffer containing β-mercaptoethanol and immediately stored at -80°C. For RNA extraction, samples were thawed and processed with the NucleoSpin^®^ RNA II kit (MACHERY-NAGEL, Düren, Germany) according to the manufacturer’s protocol. The resulting RNA was eluted with 45 µl H_2_O and concentration as well as purity was measured with a NanoDrop^®^ ND-1000 spectrophotometer (NanoDrop, Wilmington, Delaware, USA). To assess RNA integrity, 1.1% agarose gel electrophoresis was used. RNA was frozen in liquid nitrogen and stored at -80°C until further analysis.

#### 2.5.2 cDNA synthesis and RT-qPCR

Reverse transcription was conducted with 40 ng of RNA per sample using the Reverse Transcription Master Mix Kit (Fluidigm Corporation, San Francisco, California, USA), following the manufacturer’s instructions. The resulting cDNA was preamplified with 12 thermal cycles using the Fluidigm Preamp Master Mix and Delta Gene Assays according to manufacturer’s protocol. The samples were then stored at -20°C until further processing. The preamplified products were treated with exonuclease I (New England Biolabs, Ipswich, Massachusetts, USA) and subsequently diluted 10-fold with DNA suspension buffer (TEKnova, Hollister, California, USA). Bovine-specific primer pairs were designed using the National Center for Biotechnology Information (NCBI) Primer-BLAST tool. A total of 88 target genes and 8 reference genes were selected with their specifications provided in **Supplementary Table S1**. RT-qPCR was performed using Fluidigm 96x96 Dynamic Array™ Integrated Fluid Circuits (IFC), primed with the IFC Controller HX (Fluidigm) according to the manufacturer’s guidelines. The sample mix, consisting of SsoFast™ EvaGreen^®^ Supermix (Bio-Rad), DNA Binding Dye (Fluidigm), and preamplified cDNA, was added into the sample inlets. The assay mix, containing Assay Loading Reagent (Fluidigm), DNA Suspension Buffer (Teknova), and combined forward and reverse primers, was loaded into the assay inlets of the IFC. Four inter-run calibrators per chip were used as correction factors. The chips were run on the Biomark HD real-time PCR system (Fluidigm) including melting curve analysis, using the GE 96x96 Fast PCR + Melt v2 protocol with 35 cycles.

#### 2.5.3 RT-qPCR calculations and data processing

Real-time PCR Analysis Software (version 4.5.2, Fluidigm) was used to validate the PCR cycles with the default analysis settings for qPCR and melting curve analysis. The 3 most stable reference genes for data normalization were identified from a panel of 8 genes, including ACTB, B2M, GAPDH, RPLP0, RPS9, UCHL5, UXT, and YWHAZ. Therefore, the average expression stability (M-value) was determined using the geNorm algorithm (19) in R Studio (version 2023.12.1 + 402, R version 4.3.2) with packages tidyverse and ctrlGene. ACTB, B2M, and GAPDH exhibited the lowest geNorm M values and were thus selected for normalization of Cq values. Cq values were processed according to Hellemans et al. (20) to generate calibrated normalized relative quantities (CNRQ) using base R with package tidyverse and Bioconductor package HTqPCR. A total of 75 out of 88 target genes were evaluable.

### 2.6 Mitochondrial functionality of PBMC

**Figure 1:**
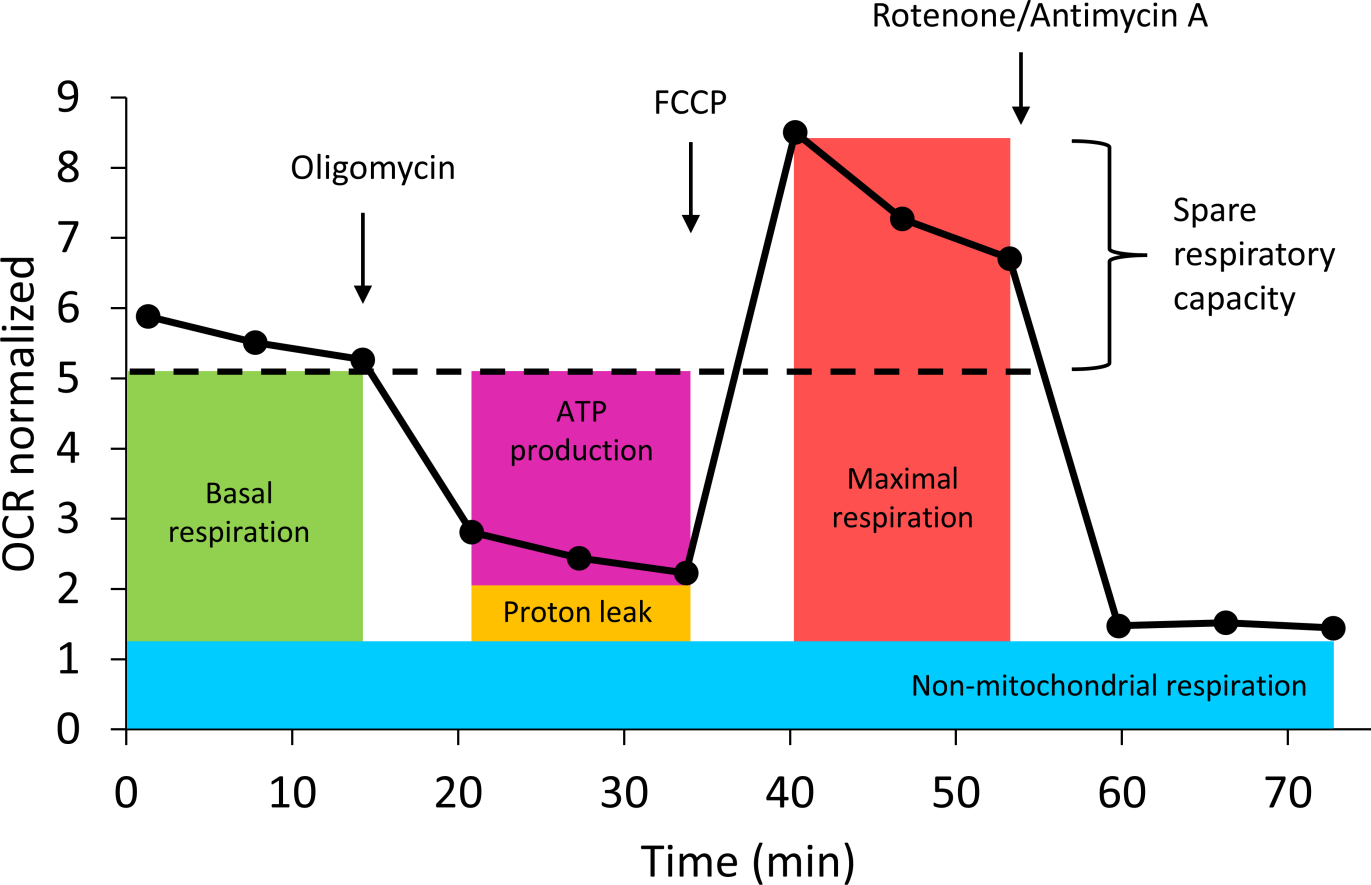
Injection strategy of Mito Stress Test (Agilent) and its effect on the oxygen consumption rate (OCR) of peripheral blood mononuclear cells from dairy cows measured with Seahorse analyzer. Oligomycin = ATP-synthase inhibitor; fluorocarbonyl cyanide phenylhydrazone = FCCP, decoupler; Rotenone/Antimycin A = complex 3/1 inhibitor. Data are shown as means of all groups, time points and stimuli (N = 345).

Mitochondrial functionality was assessed in isolated PBMC using the Seahorse XFe96 analyzer (Agilent, Santa Clara, California, USA). Immediately after density gradient centrifugation, 500,000 cells per well were seeded onto cell culture plates coated with poly-L-lysine (0.01%, Sigma-Aldrich, St. Louis, Missouri, USA). After incubation (10 min, 39°C), the plates were centrifuged (200 x g, 5 min, 20°C, Universal 320, Hettich, Tuttlingen, Germany) and the medium was replaced with pre-warmed XF Seahorse medium supplemented with glucose, sodium pyruvate and glutamine (all purchased from Agilent, Santa Clara, California, USA). Depending on the plate layout, either LPS (1 µg/ml) or ConA, (2.5 µg/ml, Sigma-Aldrich, Steinheim, Germany) was added in four replicates per cow in addition to four unstimulated wells (with Seahorse medium for volume compensation). Cell culture plates were then incubated at 39°C for 60 min without CO_2_ before measuring oxygen consumption rate (OCR) and extracellular acidification rate (ECAR) in the Seahorse analyzer. For this measurement, the sensor cartridge was hydrated the day before with XF Calibrant (Agilent, Santa Clara, California, USA) and incubated overnight (39°C, without CO_2_). OCR was recorded three times under basal conditions and three times after the addition of Mito Stress compounds (Mito Stress Test Kit, Agilent, Santa Clara, California, USA): oligomycin (oligo, 1 µM, adenosine triphosphate (ATP)-synthase inhibitor), fluorocarbonyl cyanide phenylhydrazone (FCCP, 0.5 µM, decoupler), and rotenone/antimycin A (rot/AA, 0.5 µM, complex 3/1 inhibitor), as shown in **Figure 1**. Measurements were validated using the Wave software (Agilent, Santa Clara, California, USA) and normalized to DNA concentrations per well, analyzed in stained cells (Hoechst 33342) using a plate reader (Tecan Infinite^®^ M200, Tecan Group Ltd., Männedorf, Switzerland) with an excitation wavelength of 350 nm and an emission wavelength of 461 nm. For technical reasons at 14 d *pi*, the animal number was half that of the other time points. Calculations of key parameters of the mitochondrial function were performed, consisting of basal respiration (BR, OCR_initial_ – OCR_rot/AA_), ATP production (ATP, OCR_initial_ - OCR_oligo_), proton leak (PL, OCR_oligo_ – OCR_rot/AA_), maximal respiration (MR, OCR_FCCP_ – OCR_rot/AA_), spare respiratory capacity (SRC, OCR_FCCP_ - OCR_initial_), spare respiratory capacity [%] (SRCP, MR/BR*100), non-mitochondrial respiration rate (NMRR, OCR_rot/AA_), and coupling efficiency [%] (CE, ATP/BR*100). In addition, the bioenergetic health index (BHI) (21) was calculated using the following formula: BHI = log (SRC*ATP)/(NMRR*PL). For insights in glycolytic metabolism, basal acidification (ECAR_initial_), glycolytic reserve (ECAR_oligo_ - ECAR_initial_), and compensatory glycolysis (ECAR_rot/AA_ - ECAR_initial_) were determined.

### 2.7 Calculations and statistics

All data were statistically analyzed using the MIXED procedure of SAS version 9.4 (SAS Institute Inc., Cary, North Carolina, USA) with the restricted maximum likelihood method. In the case of ConA-stimulated proliferation, intracellular carnitine and gene expression, the model included group (CON or CAR), time (relative to LPS injection), and their interaction as fixed factors. For mitochondrial functionality, the model included group (CON or CAR), time (relative to LPS injection), stimulus (unstimulated, ConA or LPS) and their interactions as fixed factors. In the model of intracellular carnitine levels, the values of d 143 *ai* were considered as covariates. The appropriate covariance structure (compound symmetry, autoregressive, variance components, or unstructured) was selected, based on the lowest Akaike information criterion (AICC). All subsequent values are presented as least square (LS) means with additional standard errors. Statistical effects were considered significant at p < 0.05. Furthermore, comparisons of LS means were performed using Tukey’s t-test. Pearson’s correlation between carnitine and its derivatives analyzed in the present investigation in PBMC and erylysate and the corresponding concentrations in plasma (6) were calculated using R Studio (version 2023.12.1 + 402, R version 4.3.2). Log_2_ fold changes were calculated for each gene between the time points and visualized with R Studio using package ComplexHeatmap (22, 23).

## 3 Results

### 3.1 Intracellular carnitine and derivatives in PBMC and erylysate

**Figure 2:**
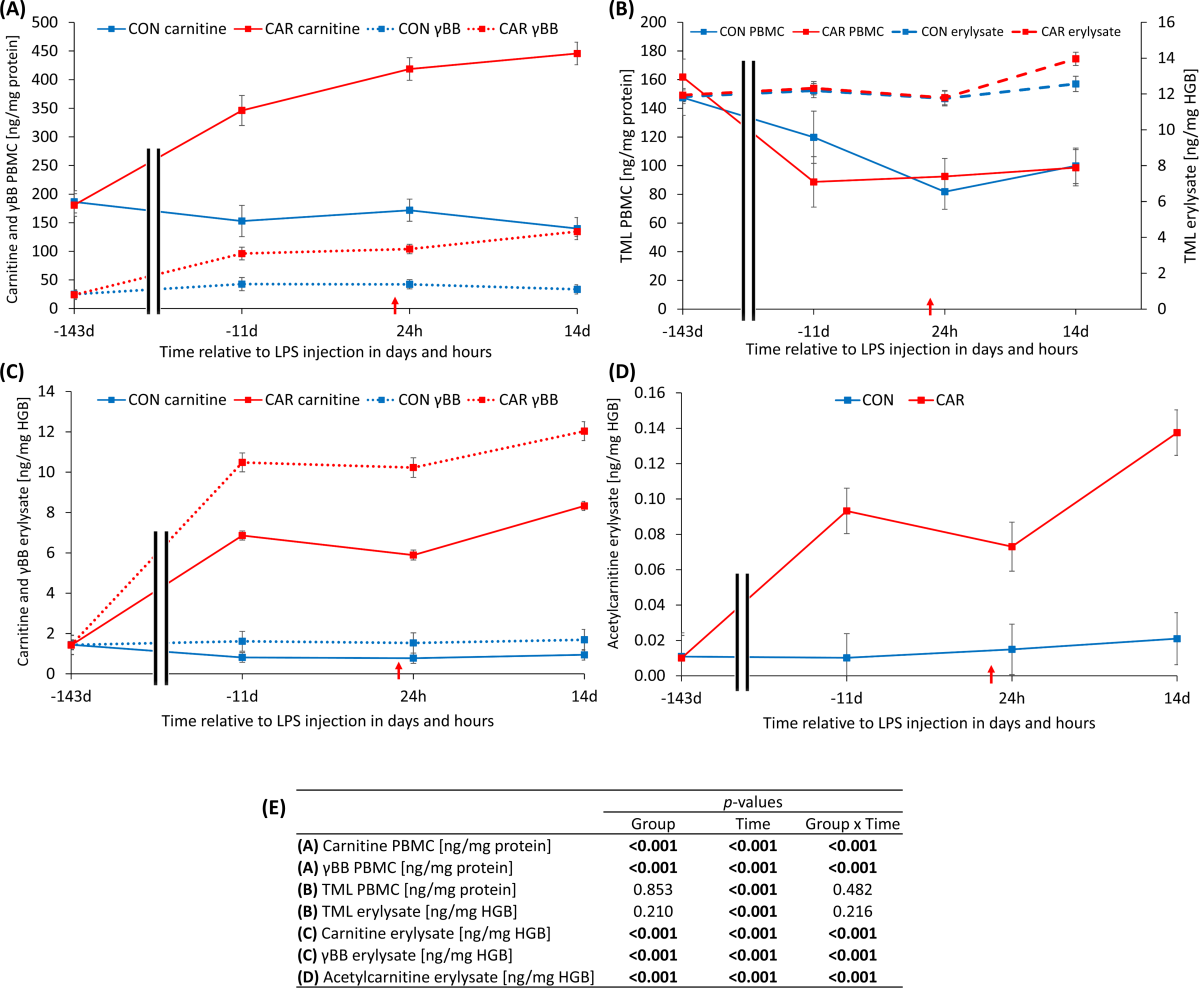
Effects of dietary L-carnitine supplementation (control group = CON; carnitine group = CAR) from 143 days before to 14 days after intravenous LPS injection (red arrow) on intracellular levels of carnitine and its derivatives of peripheral blood mononuclear cells (PBMC) and erylysate from dairy cows determined by tandem mass spectrometry. Data are presented as least square means ± standard errors. **(A)** intracellular carnitine and γ-butyrobetaine (γBB) in PBMC, **(B)** intracellular trimethyllysine (TML) in PBMC and erylysate, **(C)** intracellular carnitine and γBB in erylysate, **(D)** intracellular acetylcarnitine in erylysate. **(E)** Data statistics. HGB, hemoglobin.

All intracellular levels of carnitine and its derivatives, except for TML, were significantly affected by L-carnitine supplementation over time (p_G*T_ < 0.001). Before the start of supplementation, the carnitine concentration in PBMC (**Figure 2A**) showed no difference between the groups. Thereafter, CAR increased significantly until d 11 *ai* and subsequently more slightly until the end of the study. In comparison, CON remained at the baseline level throughout the trial. The γBB levels in PBMC (**Figure 2A**) followed the same pattern as carnitine concentrations in PBMC. Independent of L-carnitine supplementation, TML varied significantly over time (p_T_ < 0.001) in both PBMC and erylysate (**Figure 2B**). Before the supplementation, TML concentration in PBMC was significantly higher, followed by a 48% decrease until d 11 *ai*. The lower level was maintained for the rest of the study. In contrast, TML in erylysate (**Figure 2B**) remained at its initial level until 24 h *pi* and peaked at d 14 *pi*. Initially, carnitine concentration in erylysate (**Figure 2C**) was statistically equal in both groups at d 143 *ai*. Subsequently, CON did not show significant deviations from baseline throughout the study, while CAR rose to a higher level by d 11 *ai*, reaching its maximum at 14 d *pi*. γBB in erylysate (**Figure 2C**) and acetylcarnitine in erylysate (**Figure 2D**) followed a similar course as carnitine in erylysate. In PBMC, acetylcarnitine concentrations were below the detection limit. Data statistics are shown in **Figure 2E**.

**Figure 3:**
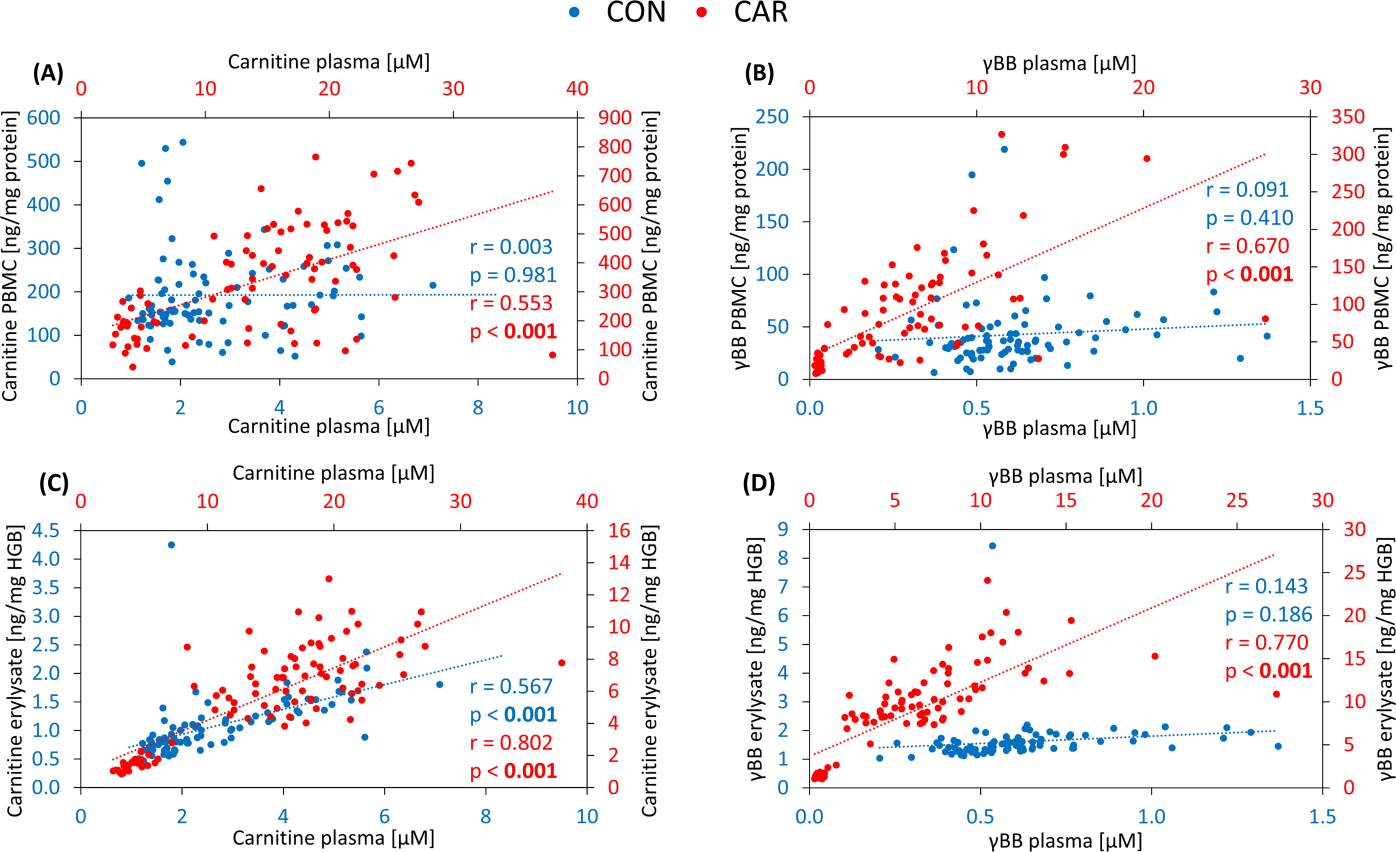
Pearson’s correlation of intracellular carnitine and γ-butyrobetaine (γBB) in peripheral blood mononuclear cells (PBMC) and erylysate with the corresponding concentrations in plasma (published in (16)) from dairy cows during dietary L-carnitine supplementation (control group = CON; carnitine group = CAR). Data are presented as individual measurements. **(A)** intracellular carnitine in PBMC relative to plasma levels, **(B)** intracellular γBB in PBMC relative to plasma levels, **(C)** intracellular carnitine in erylysate relative to plasma levels, **(D)** intracellular γBB in erylysate relative to plasma levels determined by tandem mass spectrometry. r, Pearson correlation coefficient; HGB, hemoglobin.

The data analysis revealed no correlation (r = 0.003, p = 0.981) between intracellular carnitine in PBMC and plasma carnitine concentration [published in (6)] in CON (**Figure 3A**). In contrast, a moderate positive correlation (r = 0.553, p < 0.001) was observed between these variables in CAR. A similar relationship was found for γBB levels in PBMC and plasma (**Figure 3B**). While no correlation was identified for CON (r = 0.091, p = 0.410), CAR showed a moderate positive correlation (r = 0.670, p < 0.001) between these values. Remarkably, intracellular carnitine in erylysate was moderately correlated (r = 0.567, p < 0.001) with plasma levels in CON (**Figure 3C**), and a strong positive correlation (r = 0.802, p < 0.001) was found in CAR. For γBB concentrations in erylysate, no correlation (r = 0.143, p = 0.186) with plasma levels was observed in CON, while CAR exhibited a strong positive correlation (r = 0.770, p < 0.001) between these compartments (**Figure 3D**).

**Figure 4:**
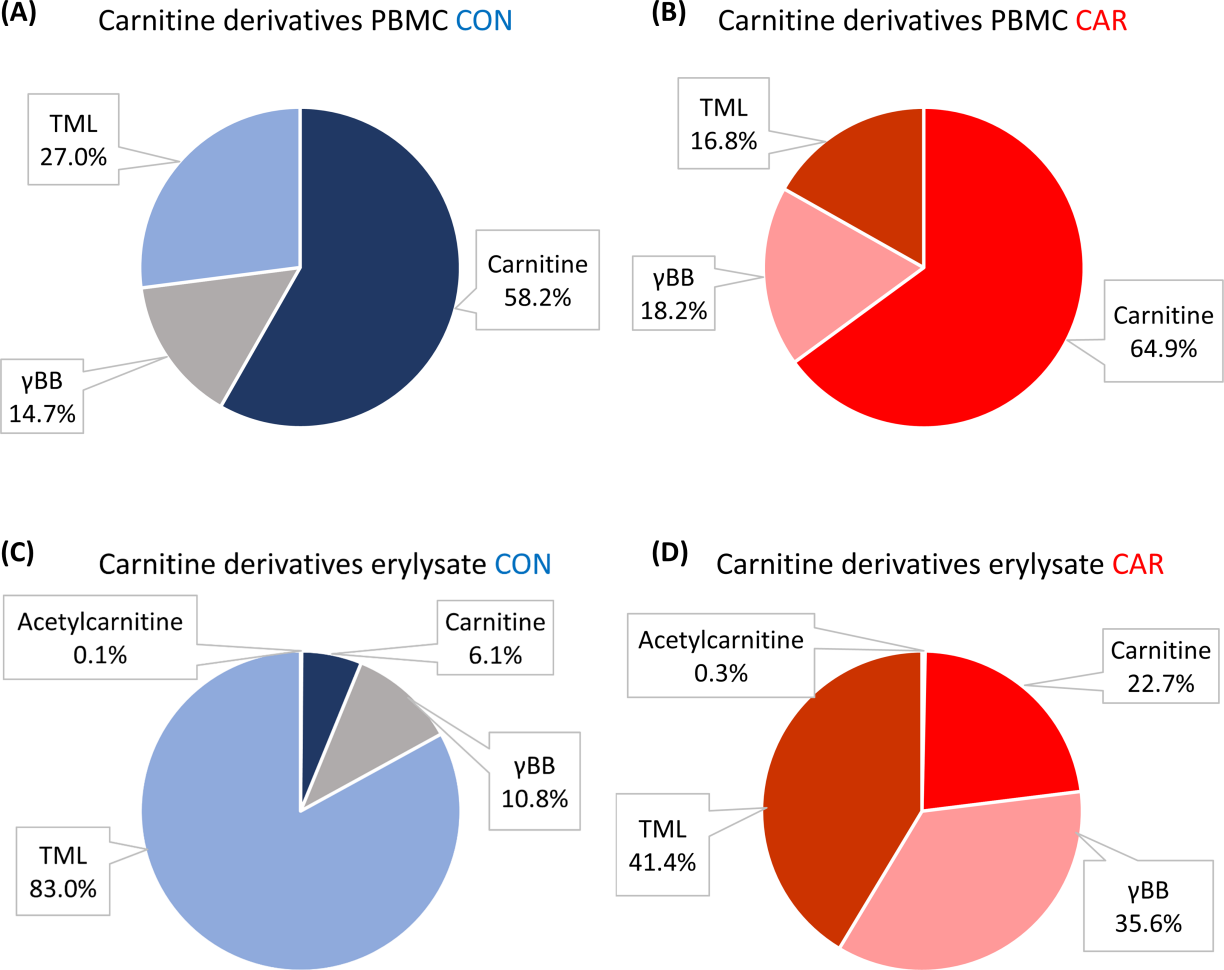
Pattern of intracellular carnitine and its derivatives in peripheral blood mononuclear cells (PBMC) and erylysate from dairy cows during dietary L-carnitine supplementation (control group = CON; carnitine group = CAR). Data are presented as LS-means ± standard error of the percentages of the sum of carnitine and its derivatives of time points -11 d, 24 h and 14 d. **(A)** intracellular carnitine and its derivatives of CON in PBMC, **(B)** intracellular carnitine and its derivatives of CAR in PBMC, **(C)** intracellular carnitine and its derivatives of CON in erylysate, **(D)** intracellular carnitine and its derivatives of CAR in erylysate determined by tandem mass spectrometry. Group differences were significant for all analyzed derivatives in both, PBMC and erylysate (p_G_ < 0.001). TML, trimethyllysine; γBB, γ-butyrobetaine.

A comparison of the pattern of intracellular carnitine and its derivatives in PBMC and erylysate, as well as between both groups, revealed notable differences. The data is described as percentages of the sum of carnitine and its derivatives of the time points -11 d, 24 h and 14 d. In PBMC (**Figures 4A, B**), intracellular carnitine was the predominant fraction (55.1% in CON and 66.1% in CAR), followed in CON by TML (31.1%) and then by γBB (13.8%), whereas in CAR it was followed by γBB (18.0%) and then by TML (15.8%). In erylysate (**Figures 4C, D**), however, the intracellular TML fraction was the most substantial (82.8% in CON and 41.9% in CAR). γBB in the erylysate of CON was similar to that in PBMC at 10.9%, while it was approximately twice as high in the erylysate of CAR (34.9%) compared to the corresponding PBMC. In erylysate of CON intracellular carnitine was 48.9 percentage points lower than in corresponding PBMC, whereas in erylysate of CAR intracellular carnitine was 43.2 percentage points lower than in corresponding PBMC. Intracellular acetylcarnitine in the erylysate of CON was 0.1% and was 3 times higher in the erylysate of CAR.

### 3.2 ConA-stimulated proliferation of PBMC

**Figure 5:**
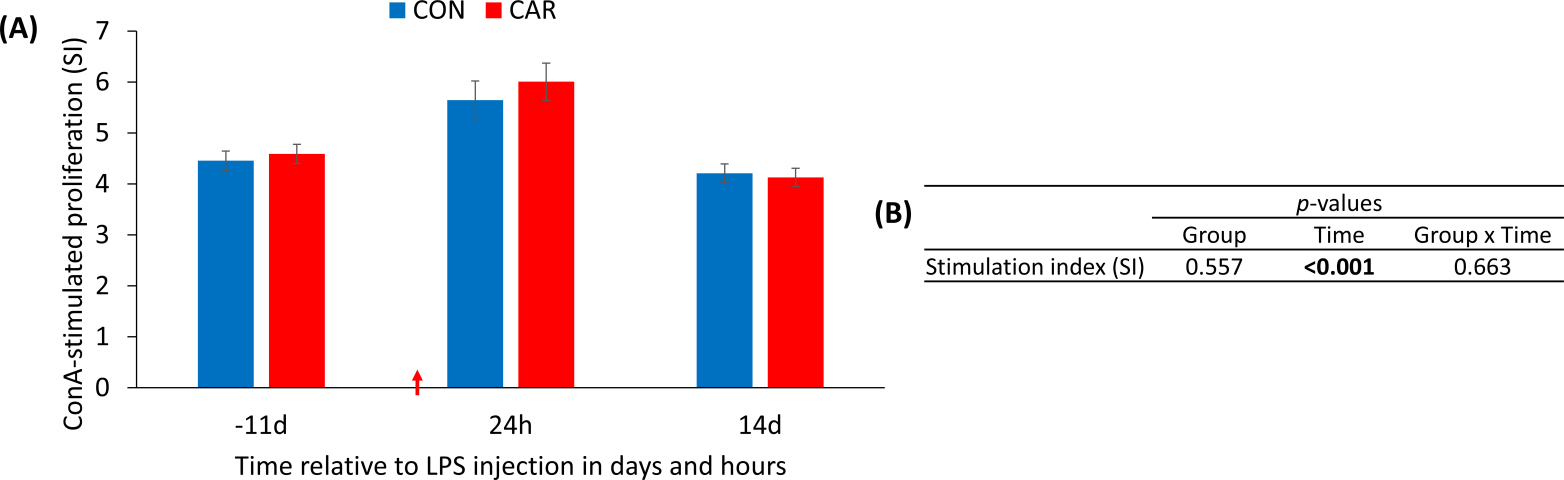
Effects of dietary L-carnitine supplementation of dairy cows (control group = CON; carnitine group = CAR) from 11 days before to 14 days after intravenous LPS injection (red arrow) on concanavalin A (ConA)-stimulated proliferation of peripheral blood mononuclear cells (PBMC). Data are presented as least square means ± standard errors. **(A)** stimulation index (SI) calculated as the ratio of fluorescence in Alamar Blue assay of ConA-stimulated to unstimulated PBMC. **(B)** Data statistics.

Independent of L-carnitine supplementation, ConA-stimulated PBMC proliferation (**Figure 5A**) was significantly affected by LPS injection (p_T_ < 0.001). The SI increased by 29% at 24 h *pi* before returning to baseline level at the end of the study. Data statistics are shown in **Figure 5B**.

### 3.3 Gene expression of PBMC

**Figure 6:**
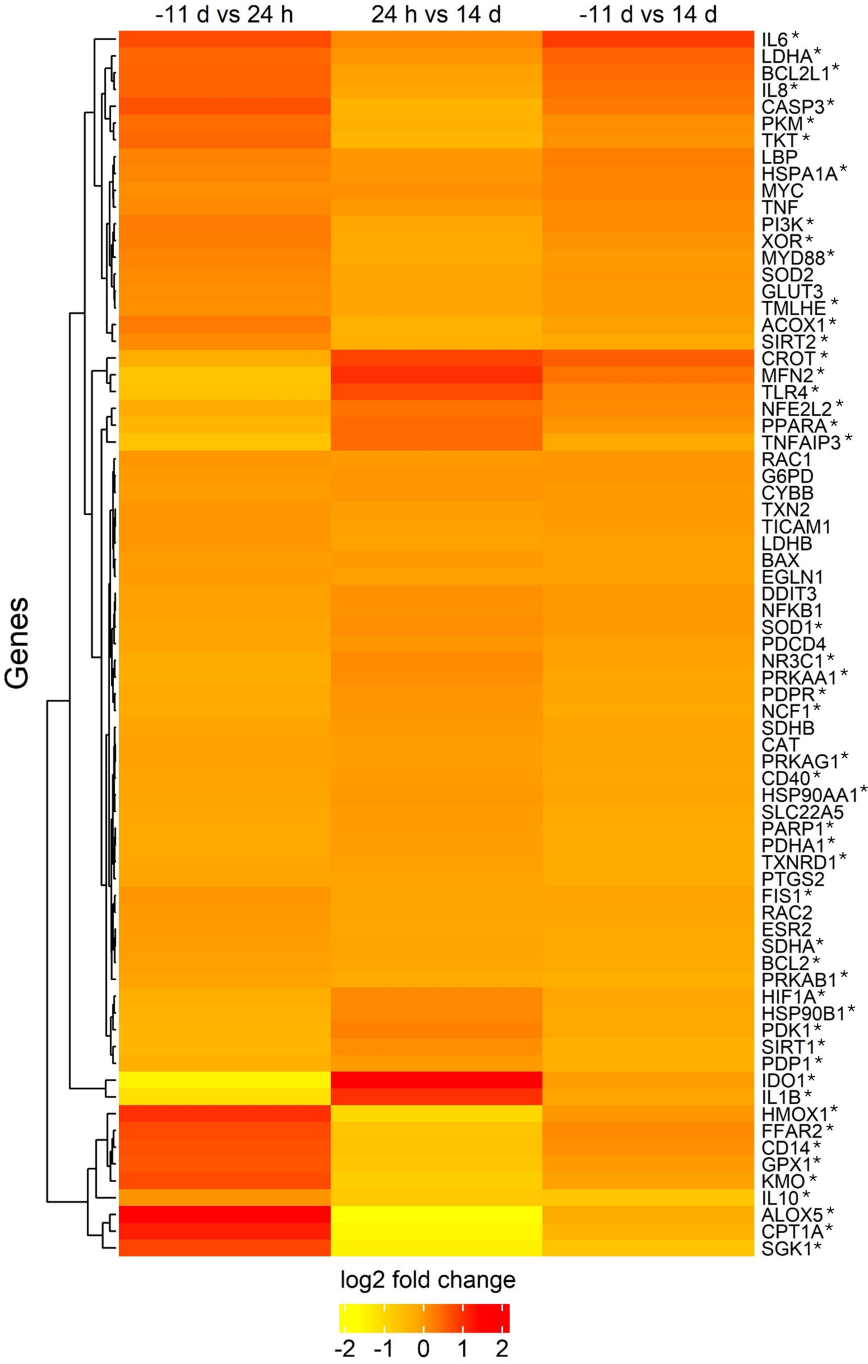
Heatmap illustrating log_2_ fold changes of calibrated normalized relative quantities of gene expression in peripheral blood mononuclear cells from dairy cows, measured with real-time qPCR. Colors represent the magnitude and direction of expression changes, with upregulated genes shown in red and downregulated genes shown in yellow. Each row corresponds to a specific gene (see **Supplementary Table S1** for details), and each column represents the comparison between two time points, irrespective of feeding groups. Rows were clustered using Euclidean distance and complete linkage. Data are presented as log_2_ fold changes of least square means. *p_Time_ < 0.05.

The log_2_ fold changes of CNRQ were visualized in a heatmap (**Figure 6**) to show the differences in gene expression between the time points (see **Supplementary Table S1** for details). The genes displayed in the heatmap were unaffected by L-carnitine supplementation. However, the expression levels of the majority of these genes was significantly influenced by LPS injection (p_T_ < 0.05), with the exception of SLC22A5, G6PD, LDHB, GLUT3, CAT, LBP, PTGS2, TNF, MYC, NFKB1, TICAM1, ESR2, CYBB, EGLN1, RAC1, RAC2, SOD2, TXN2, SDHB, BAX, DDIT3 and PDCD4 (**Supplementary Table S2**). The genes HMOX1, FFAR2, CD14, GPX1, KMO, IL10, ALOX5, CPT1A, and SGK1 showed similar expression patterns and were clustered together with upregulation initially after LPS injection and subsequent downregulation. Conversely, IDO1 and IL1B were identified as being downregulated 24 h after the immune challenge and subsequently upregulated.

**Figure 7:**
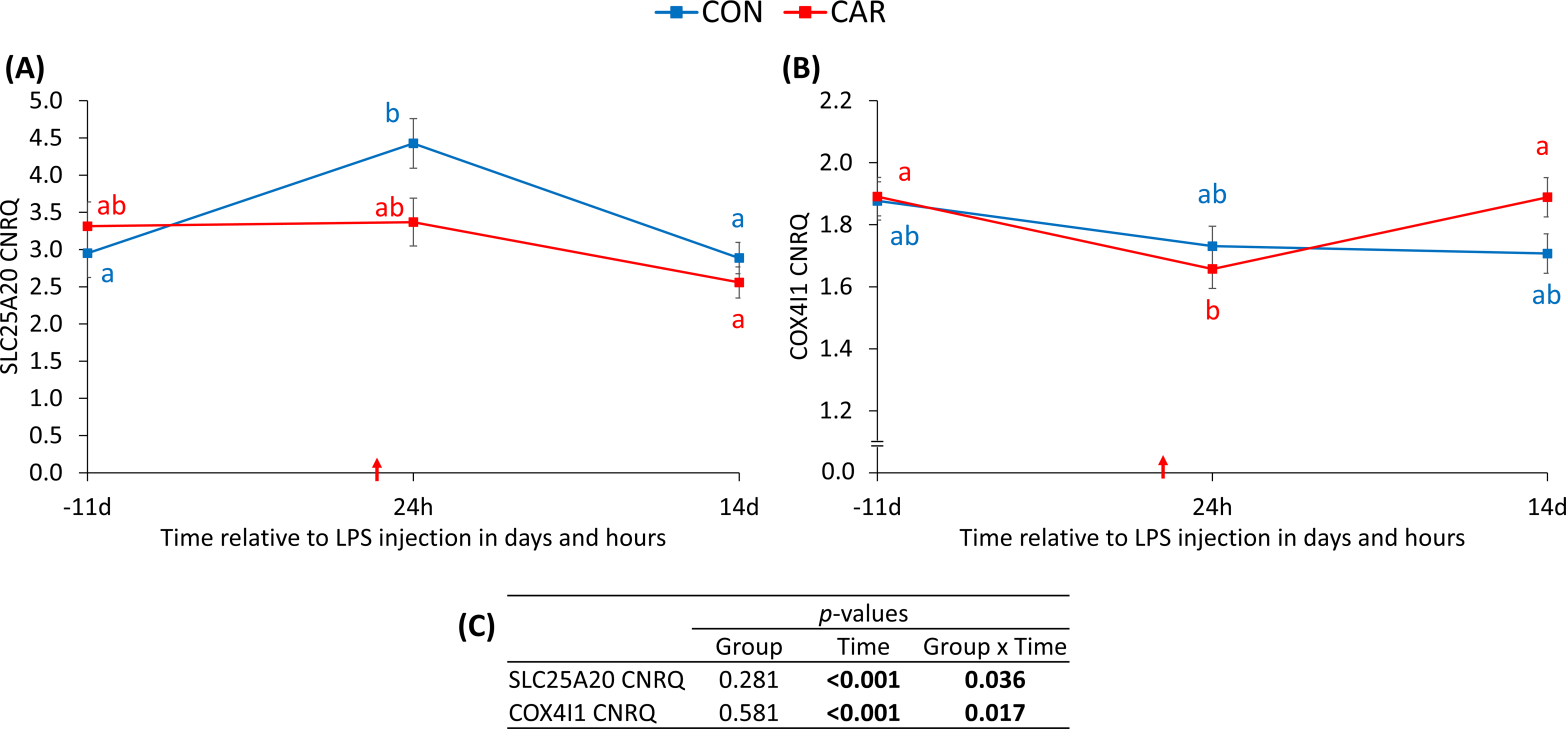
Effects of dietary L-carnitine supplementation (control group = CON; carnitine group = CAR) of dairy cows on calibrated normalized relative quantities (CNRQ) of gene expression of peripheral blood mononuclear cells from 11 days before to 14 days after intravenous LPS injection (red arrow). Data are presented as least square means ± standard errors. **(A)** CNRQ of solute carrier family 25 member 20 (SLC25A20), **(B)** CNRQ of cytochrome c oxidase subunit 4I1 (COX4I1) measured with real-time qPCR. **(C)** Data statistics. a, b different letters indicate significant differences separated by Tukey’s t-test.

The CNRQ of solute carrier family 25 member 20 (SLC25A20) was significantly affected by the interaction of group and time (p_G*T_ = 0.036, **Figure 7A**). The CNRQ of SLC25A20 remained at the baseline level throughout the trial in CAR, whereas the value increased significantly after LPS injection and returned to the initial level 14 d after immune challenge in CON. Furthermore, the CNRQ of cytochrome c oxidase subunit 4I1 (COX4I1) was also affected by the interaction of L-carnitine supplementation and LPS injection (p_G*T_ = 0.017, **Figure 7B**). CON maintained the initial level until the end of the study, while CAR decreased to the minimum at 24 h and subsequently reached the baseline level by the end of the experiment. Data statistics are shown in **Figure 7C**.

### 3.4 Mitochondrial functionality of PBMC

**Figure 8:**
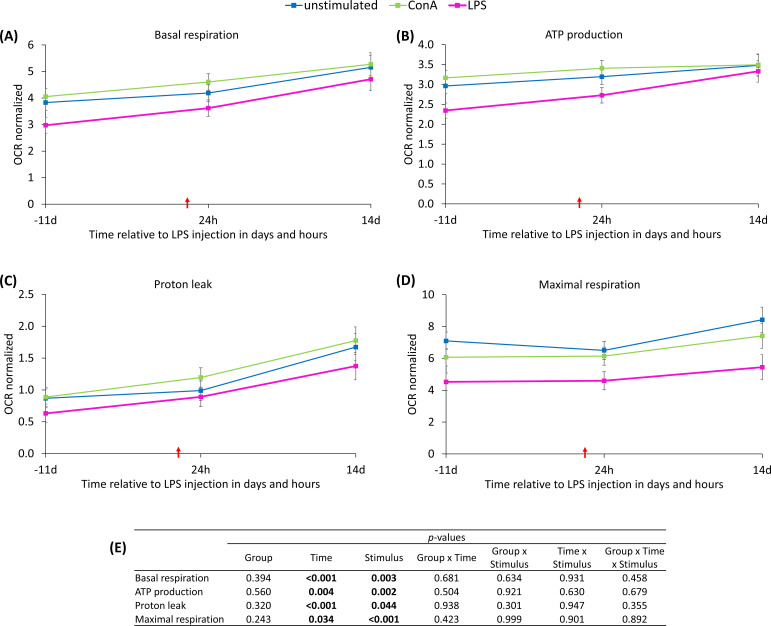
Effects of *ex vivo* stimulus (unstimulated, concanavalin A = ConA or LPS) and dietary L-carnitine supplementation of dairy cows on key parameters of mitochondrial functionality of peripheral blood mononuclear cells from 11 days before to 14 days after intravenous LPS injection (red arrow). Data are presented as least square means ± standard errors. **(A)** Oxygen consumption rate (OCR) of basal respiration, **(B)** OCR of ATP production, **(C)** OCR of proton leak, **(D)** OCR of maximal respiration measured with Seahorse analyzer and normalized to DNA content. **(E)** Data statistics.

A significant time-dependent variation (p_T_ ≤ 0.034) was found for all key parameters of mitochondrial functionality, with the exception of spare respiratory capacity and its percentage. Independent of L-carnitine supplementation, basal respiration (**Figure 8A**) was significantly higher 14 d after *in vivo* LPS administration than before and 24 h after the injection. ATP production (**Figure 8B**) increased significantly by 22% from baseline to 14 d *pi* and was not affected by group. Regarding the proton leak (**Figure 8C**), a markedly higher OCR was observed at the end of the study in comparison to the other time points. Maximal respiration (**Figure 8D**) was significantly higher at 14 d *pi* compared to 24 h *pi*. The OCR of basal respiration, ATP production, proton leak and maximal respiration was additionally affected by the *ex vivo* stimulus (p_S_ ≤ 0.044). For all of these variables, except for the proton leak, *ex vivo* LPS stimulation was significantly different from unstimulated and ConA-stimulated PBMC. The proton leak of LPS-stimulated PBMC differed significantly from ConA-stimulated PBMC, but not from unstimulated cells. Data statistics are shown in **Figure 8E**. The OCR of spare respiratory capacity and its percentage (**Supplementary Table S3**) were significantly higher in unstimulated cells compared to both types of stimulated PBMC. Furthermore, e*x vivo* LPS-stimulated cells showed a significantly lower OCR for spare respiratory capacity compared to ConA-stimulated PBMC. Irrespective of L-carnitine supplementation and *ex vivo* stimulation, significantly decreased values for coupling efficiency and bioenergetic health index (**Supplementary Table S3**) were detected 14 d after the *in vivo* immune challenge compared to the first two time points.

**Figure 9:**
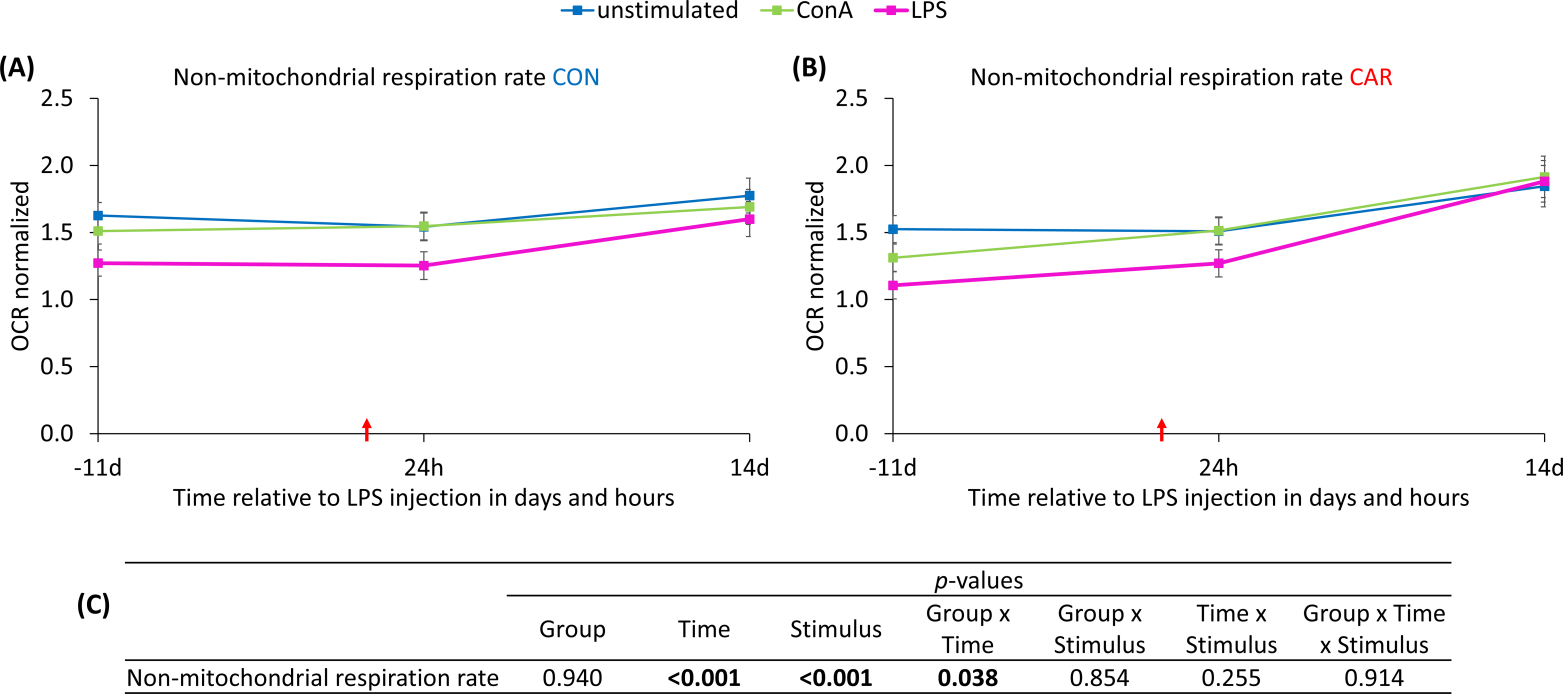
Effects of *ex vivo* stimulus (unstimulated, concanavalin A = ConA or LPS) and dietary L-carnitine supplementation (control group = CON; carnitine group = CAR) of dairy cows on non-mitochondrial respiration rate of peripheral blood mononuclear cells from 11 days before to 14 days after intravenous LPS injection (red arrow). Data are presented as least square means ± standard errors. **(A)** Oxygen consumption rate (OCR) of non-mitochondrial respiration rate of CON, **(B)** OCR of non-mitochondrial respiration rate of CAR measured with Seahorse analyzer and normalized to DNA content. **(C)** Data statistics.

Having a closer look at the non-mitochondrial respiration rate, a significant interaction between group and time was observed (p_G*T_ = 0.038), in addition to the effect of the *ex vivo* stimulus. For this variable, *ex vivo* LPS-stimulated cells showed a lower OCR, compared to the others. In CON, the non-mitochondrial respiration rate (**Figure 9A**) remained at the baseline level throughout the experiment. In contrast, CAR (**Figure 9B**) showed a significantly higher OCR at 14 d after LPS injection compared to 11 d *ai* and 24 h *pi*, resulting in the significant interaction. Data statistics are shown in **Figure 9C**.

**Figure 10:**
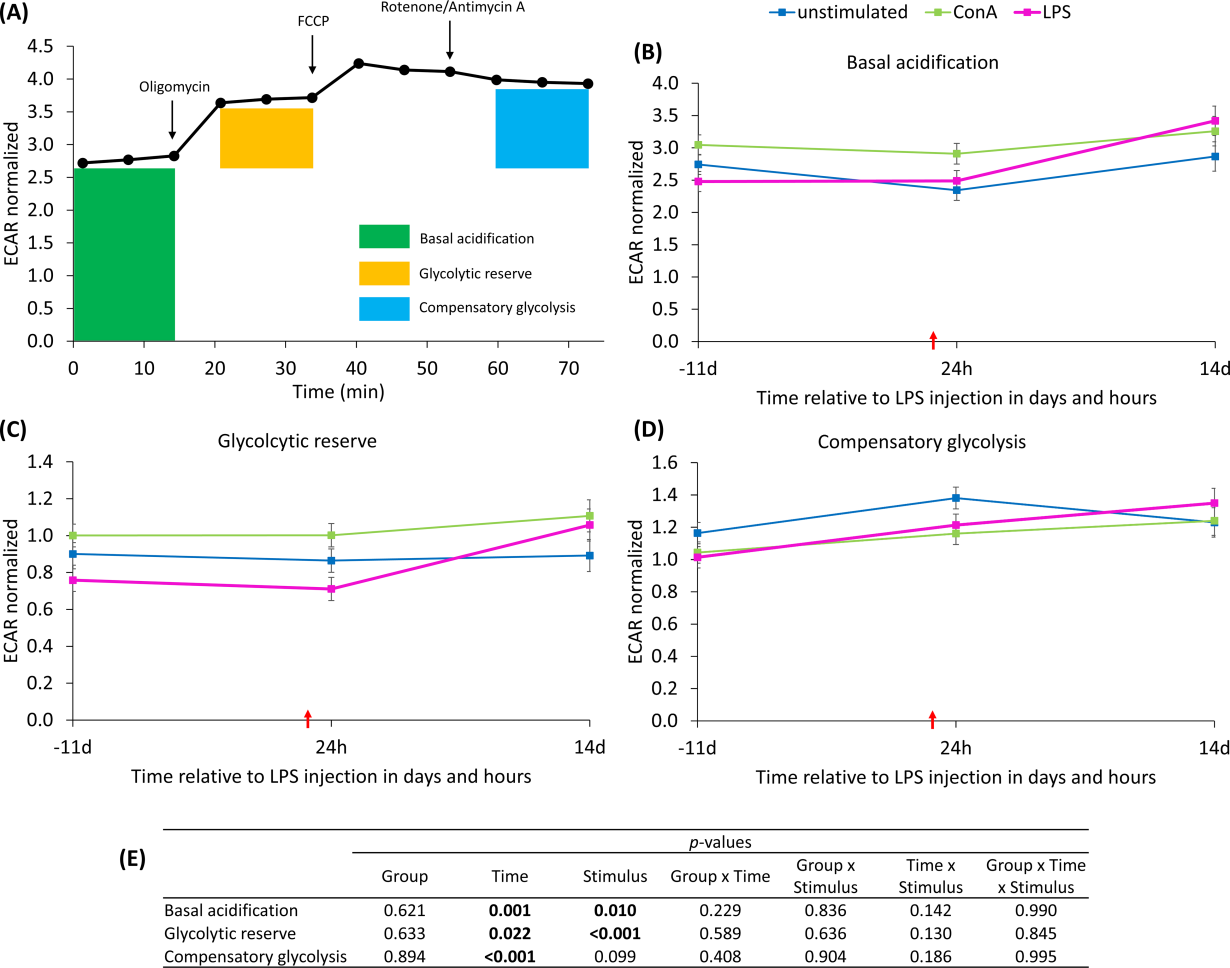
**(A)** Injection strategy of Mito Stress Test (Agilent) and its effect on the extracellular acidification rate (ECAR) of peripheral blood mononuclear cells from dairy cows measured with Seahorse analyzer. Oligomycin = ATP-synthase inhibitor; fluorocarbonyl cyanide phenylhydrazone = FCCP, decoupler; Rotenone/Antimycin A = complex 3/1 inhibitor. Data are shown as means of all groups, time points and stimuli (N = 345). **(B–E)** Effects of ex vivo stimulus (unstimulated, concanavalin A = ConA or LPS) and dietary L-carnitine supplementation of dairy cows on key parameters of glycolytic metabolism of peripheral blood mononuclear cells from 11 days before to 14 days after intravenous LPS injection (red arrow). Data are presented as least square means ± standard errors. **(B)** ECAR of basal acidification, **(C)** ECAR of glycolytic reserve, **(D)** ECAR of compensatory glycolysis measured with Seahorse analyzer and normalized to DNA content. **(E)** Data statistics.

An overview of the ECAR calculations is given in **Figure 10A**. The basal acidification (**Figure 10B**) and glycolytic reserve (**Figure 10C**) remained at the basal level 24 h *pi* and increased significantly at 14 d *pi*. In contrast, the compensatory glycolysis (**Figure 10D**) already increased at 24 h *pi* and maintained the higher level until the end of the trial. Data statistics are shown in **Figure 10E**.

## 4 Discussion

The aim of the present study was to examine the influence of dietary L-carnitine supplementation on the metabolism of PBMC in mid-lactating dairy cows undergoing LPS-induced systemic inflammation. A number of studies have investigated the influence of L-carnitine supplementation in cattle (24–26). However, none of these studies specifically addressed the impact of such supplementation on PBMC metabolism during an inflammatory challenge.

In ruminants, a number of regulatory mechanisms are involved in the maintenance of carnitine homeostasis (27). As previously published by Meyer et al. (6), the mean plasma carnitine concentration in this trial was seven times higher in L-carnitine supplemented cows compared to the control group, indicating effective absorption from the gastrointestinal tract to the bloodstream. Additionally, plasma γBB levels were significantly higher in CAR compared to CON (6), showing that supplementation raises both carnitine and its precursor γBB. The transport of carnitine and γBB (27) from the extracellular to the intracellular environment is mainly facilitated by the organic cation transporter novel 2 OCTN2 (28, 29), also known as solute carrier family 22 member 5 (SLC22A5), and by OCTN1 (29). Pochini et al. (30) reviewed that OCTN2 and OCTN1 are integrated in the membrane of immune cells, and PCR analysis confirmed the presence of SLC22A5 in bovine PBMC in the current study. In CAR, intracellular concentrations of carnitine and γBB in PBMC progressively increased until the end of the study, suggesting an accumulation in PBMC over time. Moreover, higher plasma carnitine and γBB levels resulted in higher intracellular carnitine levels in PBMC of supplemented cows. The lack of upregulation of SLC22A5 at the gene level in CAR suggests that existing transporter capacities in bovine PBMC were adequate to handle the higher substrate flux from the extracellular to the intracellular space. The missing correlation of carnitine and γBB levels between plasma and PBMC in CON may be attributable to a lower probability of transport to the intracellular side at physiologically lower plasma levels.

The carnitine biosynthesis is, among other things (27), regulated by negative feedback mechanisms. Elevated plasma carnitine levels likely inhibit endogenous production by suppressing γ-butyrobetaine hydroxylase, the enzyme which converts γBB to carnitine (5), therefore leading to an accumulation of γBB. Additionally, the results of our study suggest that the conversion of TML by the enzyme ϵ-N-trimethyllysine hydroxylase and the synthesis of TML itself (5) are not affected by higher plasma carnitine levels. This observation coincides with *in vitro* findings by Rebouche et al. (31), where higher L-carnitine concentrations in the medium inhibited γ-butyrobetaine hydroxylase activity but left ϵ-N-trimethyllysine hydroxylase activity unaffected. This could explain the missing effect of L-carnitine supplementation on TML plasma (6) and intracellular levels.

Mammalian erythrocytes do not have cell organelles (32) and are therefore incapable of mitochondrial β-oxidation. Nevertheless, carnitine and its derivatives were detected in erythrocytes in the present study, as already described by Cooper et al. (33). Since there is no documented evidence in the literature for the presence of OCTN1 and OCTN2 transporters in the membrane of erythrocytes, a possible way of carnitine transfer might be passive diffusion (34). In contrast to the findings of Cooper et al. (33), who observed a significantly higher concentration of intracellular acetylcarnitine compared to carnitine in human erythrocytes, our results demonstrated that acetylcarnitine represented 0.1% in CON and 0.3% of all derivatives in CAR, compared to 6.2% and 22.9%, respectively, for carnitine. Additionally, Evans et al. (35) postulated a missing transport mechanism between plasma and mature erythrocytes in humans, which is contradicted in bovine erythrocytes, as the intracellular levels of carnitine and γBB in erythrocytes increased in L-carnitine supplemented cows in the present study. Furthermore, a positive correlation between plasma carnitine levels and erylysate was observed in both CON and CAR, underlining a functioning balance. The observation that γBB only showed a correlation between plasma and erylysate in CAR could give a hint that a higher concentration of γBB is required to establish balance between these compartments. The difference in distribution of carnitine and its derivatives between PBMC and erylysate in the present study with higher carnitine proportions in PBMC might be a consequence of different transport mechanisms as carnitine requirement of erythrocytes, cells without mitochondria, could be lower than in PBMC.

The increased ConA-stimulated proliferation of PBMC due to LPS injection is a good indicator of a functioning immune response and indicates that e. g. T-cells are in an activated status (36). Having a closer look at the heat map illustrating the gene expression profile of PBMC, most of the genes showed massive changes in expression due to LPS injection, but were not affected by L-carnitine supplementation. To highlight some immune-related genes, the pro-inflammatory cytokine interleukin-1β (IL-1β) was notably downregulated 24 h *pi* compared to before and 14 d after the immune challenge. Yoo et al. (37) reported that the mRNA expression of IL-1β in cultured bovine alveolar macrophages reached its maximum at 1 to 2 h following incubation with LPS from *Pasteurella haemolytica* and was not detectable 24 h after stimulation, indicating a downregulation of this cytokine. Because of the missing detectability, it remained unclear, if the level was the same or below the baseline, as observed in our study. Additionally, Caroll et al. (38) demonstrated, that serum levels of the protein IL-1β peaked at 3 h following an intravenous injection of 2.5 µg/kg LPS in steers and began to decline 4.5 h after the bolus injection. In their study, IL-1β returned to baseline within 8 h after the immune challenge suggesting a steady decrease, mediated by negative feedback mechanisms (39).

The anti-inflammatory cytokine interleukin-10 (IL-10), which also inhibits IL-1β production (40), maintained the baseline level at 24 h in the present study and was downregulated at 14 d *pi*. Although no samples were examined within the first 24 h, it can be suggested that IL-10 expression increased during this period after LPS injection, as observed in other studies. Calvano et al. (41) discovered that the gene expression of IL-10 was upregulated to the maximum in human leukocytes between 4 and 6 hours after endotoxin infusion, indicating the onset of a transition from a pro-inflammatory to an anti-inflammatory response in this period. A study by Dänicke et al. (42), in which pigs were intravenously infused with 7.5 µg LPS/kg BW/h for 1 h, showed a peak of plasma IL-10 at 1 h after LPS infusion and a subsequent decrease. These results showed an earlier peak of the anti-inflammatory IL-10 in porcine plasma compared to the gene expression of human leukocytes studied by Calvano et al. (41).

In contrast to IL-10, tumor necrosis factor α induced protein 3 (TNFAIP3), also known as an anti-inflammatory protein (43), was already downregulated at 24 h and reached again the initial level at 14 d *pi*. Therefore, the upregulation of TNFAIP3 appears to be a more rapid response to inflammation that was already below the initial level 24 h after LPS injection, whereas IL-10 was still expressed at baseline level.

The present data suggest that the acute pro-inflammatory response had already a shift to an anti-inflammatory state at 24 h *pi*. This hypothesis is supported by the study of Calvano et al. (41), who investigated gene expression profiles of human leukocytes at 2, 4, 6, 9, and 24 hours following endotoxin infusion. Their study demonstrated that acute pro-inflammatory changes were already observed 2–4 hours after the stimulus, whereas anti-inflammatory genes, including IL-10, peaked between 4 and 6 hours. Consistent with our findings in bovine PBMC, Calvano et al. (41) observed that the majority of the investigated genes in human leukocytes had recovered 24 h after an induced inflammatory challenge. The clinical status of the cows in the present study also shows a pro-inflammatory and anti-inflammatory period, according to the gene expression profile of PBMC. The cumulative clinical score rose in the first 4 h after LPS injection, which belongs to pro-inflammatory mechanisms and reached again the initial level at 9 h *pi* (6), emphasizing regulatory anti-inflammatory mechanisms. It would be beneficial for future studies to determine the gene expression of bovine PBMC at a higher frequency immediately after LPS injection in order to investigate the acute dynamics of gene expression and potential benefits of L-carnitine supplementation during this critical period.

Regarding carnitine-associated genes, the carnitine palmitoyl transferase 1 isoform a (CPT1a) was upregulated in both feeding groups at 24 h *pi*, which is consistent with the results from the mRNA abundance in hepatocytes (44). This enzyme is important for coupling LCFA and carnitine in the cytosol, facilitating their transport across mitochondrial membranes (4). After the onset of bacterial-induced inflammation, activated immune cells are known to increase their rate of glycolysis (45). However, the upregulation of CPT1a at 24 h *pi* in our study may indicate the initiation of a transition phase from the LPS-induced glycolytic shift (14), which may have occurred prior to the 24 h time point in the present study, towards β-oxidation. This is supported by the observation that basal acidification and glycolytic reserve were at basal levels at 24 h, but the compensatory glycolysis was increased at this time point. This finding suggests that the cell population may have a higher capacity to activate glycolysis compared to its state prior to the LPS challenge. Additionally, it needs to be considered that PBMC are a heterogenous population with diverse cell types. While individual PBMC subpopulations may already be capable of activating β-oxidation, a proportion of the PBMC cell population may persist in a glycolytic state. Moreover, it cannot be excluded that the activation status of immune cells may differ from that observed in natural bacterial inflammation, as the LPS injection is a research model lacking the dynamics of a bacterial infection. Further research is required to compare the gene expression of PBMC during natural systemic *E. coli* infections with those observed in the inflammatory model with LPS.

The protein carnitine-acylcarnitine translocase, also known as solute carrier family 25 member 20 (SLC25A20), is an important component of the inner mitochondrial membrane and is essential for the transport of acylcarnitine into the mitochondrial matrix (4). Acylcarnitine is exchanged for L-carnitine in a one-to-one ratio (4). The present results demonstrated an upregulation of SLC25A20 at the mRNA level in CON 24 h after LPS injection, whereas CAR maintained baseline values. This upregulation may be a compensatory adaptation to the increased energy demands associated with the immune response to LPS (46). Due to the higher availability of L-carnitine, CAR may have an adequate flux of L-carnitine and acylcarnitine in PBMC during LPS-induced inflammation and may not need to adapt by upregulating SLC25A20 in PBMC.

Cytochrome c oxidase subunit 4 (COX4I1) is part of an enzyme complex located in the inner mitochondrial membrane and belongs to the electron transfer chain (47). This gene showed a contrasting expression pattern and was downregulated in CAR by LPS injection in the present study, whereas CON maintained baseline values. In hepatocytes, the mRNA abundance of COX4I1 was not significantly affected by L-carnitine supplementation over the trial (44). Since there is no evidence in the literature for a direct or indirect effect of L-carnitine on COX4I1 and all other parameters related to the respiratory chain remained unaffected, this isolated effect cannot be plausibly explained.

The basal respiration offers insights into the basal status of oxidative phosphorylation (OXPHOS) in PBMC and comprises two components: mitochondrial ATP production and proton leak. ATP production constitutes the part of direct ATP synthesis by OXPHOS, and the proton leak defines the inefficient flux of protons through the inner mitochondrial membrane without ATP production. One day after the *in vivo* LPS challenge, PBMC showed the same level of basal respiration, ATP production, proton leak, and maximal respiration like before the challenge, indicating unaffected basal activity of OXPHOS. The significant increase in basal respiration and maximal respiration 14 d *pi* indicates an enhanced activity of the electron transport chain, potentially reflecting a higher energetic demand and mitochondrial adaptation. This activation may occur due to regeneration and reparation processes as well as mitochondrial biogenesis (48). However, the relatively modest increase in ATP production (~22%) compared to the doubling of proton leak suggests that part of the respiratory activity may be uncoupled from ATP synthesis at the end of the study. The higher proton leak may give a hint at mitochondrial adaptation to protect the cells from the formation of reactive oxygen species (ROS), potentially induced by a higher electron flux (49). To compensate for the resulting ATP loss, the PBMC increase their glycolytic activity, which is reflected in increased ECAR values of all glycolytic variables in the Mito Stress Test.

Looking more closely at the effect of the *ex vivo* stimulus, LPS stimulation resulted in a lower OCR compared to unstimulated cells for basal respiration, ATP production, and maximal respiration. All investigated ECAR parameters revealed no significant differences between unstimulated and LPS stimulated PBMC. In a study by Haschemi et al. (50), primary mouse macrophages were stimulated with LPS, and real-time OCR and ECAR were determined. In contrast to our results, the authors demonstrated an increase in ECAR within the first hour following *in vitro* LPS addition and interpreted this as indicative of a switch from oxidative phosphorylation to increased glycolysis as part of the M1 macrophage response following LPS stimulation (51). Concurrently, a decrease in OCR was shown, suggesting a decrease in the activity of the mitochondrial respiratory chain, which is consistent to our results. One of the main differences of the aforementioned study and our model is the utilized cell type. Compared to mouse macrophages, the primary bovine PBMC used in this study constituted a heterogenous cell population, comprising a monocyte population of approximately 10% as shown by hematological analyses (11, 17). Within PBMC population only monocytes are known to express Toll-like receptor 4 (TLR4) (52), which enables them to recognize LPS and respond directly to it. Consequently, the proportion of cells directly responding to LPS in the mixed population used in this study may be inadequate to demonstrate the effect on increased ECAR described in the aforementioned study. Nevertheless, it can be hypothesized, that the whole PBMC population was experiencing metabolic stress by LPS addition in the present study, which may be the reason for decreased OCR.

Non-mitochondrial respiration rate is mainly composed of oxygen consumption by cellular enzyme activity, such as oxidases (53). The reason for the increase in non-mitochondrial respiration in CAR on d 14 *pi*, compared to the steady level in CON still remains unclear, as there is no evidence in the literature for an effect of L-carnitine on these enzymes. In this study, we investigated the gene expression of the peroxisomal enzymes carnitine O-octanoyltransferase (CROT) and acyl-CoA oxidase 1 (ACOX1) which both contribute to peroxisomal β-oxidation. ACOX1 catalyzes the initial step of peroxisomal β-oxidation and produces H_2_O_2_ under O_2_ consumption that contributes to the non-mitochondrial respiration rate (54). CROT catalyzes the transport of the end product of peroxisomal β-oxidation from the peroxisome into the mitochondria (55), thereby indirectly increasing ACOX1 activity and non-mitochondrial respiration by enhancing the flux of peroxisomal β-oxidation. The gene expression of both enzymes was significantly affected by intravenous LPS injection, but not by L-carnitine supplementation. Consequently, the observed differences between both feeding groups in non-mitochondrial respiration may not be exclusively explained by this metabolic pathway. Furthermore, the results from d 14 *pi* must be interpreted with caution due to the discrepancy in sample size. In addition, all related variables in this experiment, like ROS production in PBMC and enzyme activity of superoxide dismutase and glutathione peroxidase (11), were not affected by dietary L-carnitine supplementation.

The present study showed a balanced distribution of carnitine and γ-butyrobetaine between blood cells and plasma of dairy cows during dietary L-carnitine supplementation, independent of transcriptional regulation of the transporter gene SLC22A5, indicating sufficient baseline transport capacity in PBMCs. However, the study revealed that the metabolism of PBMC was not directly supported by L-carnitine one day and two weeks after an intravenous immune challenge. This outcome is consistent with the established knowledge that activated lymphocytes prioritize glycolytic ATP generation (14), which may be one of the reasons for the missing support of L-carnitine. For future studies, it is recommended to select earlier time points and a higher frequency to assess gene expression and mitochondrial functionality of PBMC following an LPS challenge, as the primary dynamics of the immune response occur immediately after the immune challenge.

## Data Availability

Data in this manuscript were collected and managed in accordance with the data management policy of the FLI. Raw data for statistical analyses and supplemental data are available at Zenodo (DOI: 10.5281/zenodo.14916225) https://zenodo.org/records/14916225?preview=1&token=eyJhbGciOiJIUzUxMiJ9.eyJpZCI6IjE4NGQ2MjQyLTAyMmEtNDEyMy1iYjQ2LWJmZjUwMDdmMGQ2OCIsImRhdGEiOnt9LCJyYW5kb20iOiJhODQwNzZiNDI0NDdjNWJiNjIxMjVhNDhkZjQ2NTNmMCJ9.AllWNdpVipOaZDA0pcLVB3PPf5kQtUcQwAU6uGDYh5dyIWEGGNSn6NdeINGLfH3fZFZ_VYo3Jccq4PH4ks-6HQ.
