# Supplementary material for: Dietary supplementation with L-carnitine elevates intracellular carnitine levels and affects gene expression of SLC25A20 and COX4I1, as well as non-mitochondrial respiration of bovine blood cells during systemic immune challenge
Source: Front Immunol. 2025 Jul 11;16:1583351. doi: 10.3389/fimmu.2025.1583351 (PMC12289496; doi:10.3389/fimmu.2025.1583351)
Supplement: Supplementary file 1 [file DataSheet1.pdf]

# *Supplementary Material*

**Supplementary Table 1:** Details of gene-specific primers used for RT-qPCR.

| Gene symbol | Gene name | NCBI Accession number | Sequences [fw/rev] 5‘-3‘ | Amplicon size [bp] | Reference |
|---|---|---|---|---|---|
| **Carnitine associated** | | | | | |
| CPT1A | carnitine palmitoyltransferase 1A | NM_001304989.2 | F-CCTATTTTGGACACGGGAAA<br>R-TCAAACCACCTGTCGAAACA | 172 | |
| CROT | carnitine O-octanoyltransferase | NM_177494.2 | F-AGTACGCTGGGGTGACAAATC<br>R-AACAAGCTCCTCTGGAACCG | 199 | |
| SLC22A5 (OCTN2) | solute carrier family 22 member 5 | NM_001046502.2 | F-ACTCCAGTGAGCTACAAGACC<br>R-ACGTCCCCATGCAAGTTAGG | 175 | |
| SLC25A20 | solute carrier family 25 member 20 | NM_001077936.2 | F-GTTCCTTAACTGGGCTGTGC<br>R-ACAATGGTCTCGACAGGTCC | 172 | Ghaffari et al. 2021, doi:10.3168/jds.2021-20237 |
| TMLHE | trimethyllysine hydroxylase, epsilon | NM_001076064.1 | F-GGGGTTGGGCCAGTCTTAAA<br>R-GACAGCCCGGTCATAGTTGT | 81 | Ghaffari et al. 2021, doi:10.3168/jds.2021-20237 |
| **Energy metabolism** | | | | | |
| ACOX1 | acyl-CoA oxidase 1 | NM_001035289.3 | F-AGGAAGTTTGGCATCGCAGA<br>R-TAATTGAGGCCCACAGGTTCC | 89 | Ghaffari et al. 2021, doi:10.3168/jds.2021-20237 |
| G6PD | glucose-6-phosphate dehydrogenase | NM_001244135.2 | F-TCGGAGCTTGACCTGACCTA<br>R-TTCGATGTGGTGGAGCAGTG | 168 | |
| LDHA | lactate dehydrogenase A | NM_174099.2 | F-GCCTGAGAAGTCGGAGTGT<br>R-GTCCTACCTGCCCATGAATGTT | 109 | |
| LDHB | lactate dehydrogenase B | NM_174100.2 | F-GGCGTTTCTCTCCAGGAACT<br>R-GCCATACATGCCCTTCACCAT | 225 | |
| PDHA1 | pyruvate dehydrogenase E1 alpha 1 subunit | NM_001101046.2 | F-GGCTCGTTGAAGGTTCGCAG<br>R-CAAAATGACGAGATGCCACCAG | 133 | |
| PDK1 | pyruvate dehydrogenase kinase 1 | NM_001205957.1 | F-TCATCGGAAACACGTCGGAA<br>R-ATCACACAGACGCCTAGCAT | 91 | |
| PDP1 | pyruvate dehydrogenase phosphatase catalytic subunit 1 | NM_001206353.1 | F-CATGCCAGCACCAACTCAAC<br>R-CGGATGGGGTGTGTATCTCG | 151 | |
| PDPR | pyruvate dehydrogenase phosphatase regulatory subunit | NM_174781.2 | F-TGACTCCAGACCACTTCCCA<br>R-CCCCACGCTCATCACTTCAT | 164 | |
| PKM | pyruvate kinase M1/2 | NM_001205727.1 | F-AGAGGCTGCCATCTACCATTTG<br>R-GCGAGCTGTCTGGTGATTCC | 229 | |

| | | | | | |
|---|---|---|---|---|---|
| PRKAG1 | protein kinase AMP-activated non-catalytic subunit gamma 1 | NM_174586.2 | F-GATGAGAAAGGGCGTGTGGT<br>R-AGGTCTTTTCTGCTGCCAGG | 70 | Ghaffari et al. 2021, doi:10.3168/jds.2021-20237 |
| PRKAA1 | protein kinase AMP-activated catalytic subunit alpha 1 | NM_001109802.2 | F-GGGAAGCAACTGCCTAAACC<br>R-CACAGCAACTTTATGCCCAGTC | 95 | |
| PRKAB1 | protein kinase AMP-activated non-catalytic subunit beta 1 | NM_001024558.1 | F-GAGTTGTCCAGTTCCCCACC<br>R-GCACCATCACTCCATCCTTGA | 220 | |
| TKT | transketolase | NM_001003906.1 | F-TCTTTACACGGGCCTTCGAC<br>R-TACTGCCTTCTCCGTAGCCA | 200 | Heymann et al. 2021, doi:10.1371/journal.pone.0246679 |
| **Transporters and receptors** | | | | | |
| FFAR2 | free fatty acid receptor 2 | NM_001163784.1 | F-GCTGTGTGAAATGGGCGGTA<br>R-GGCATGGCTGTCCTTGTCTT | 126 | Heymann et al. 2023, doi:10.1371/journal.pone.0286995 |
| GLUT3 (SLC2A3) | solute carrier family 2 member 3 | NM_174603.3 | F-TTCCCTGGTTTATTGTGGCTGA<br>R-TTGAAGAAGGTGAAGACCCAGA | 196 | |
| **Immune response** | | | | | |
| CAT | catalase | NM_001035386.2 | F-CATTGCGGGCCATCTGAAAG<br>R-AAGTGAGACCCATGCTGCAC | 171 | |
| CD14 | CD14 molecule | NM_174008.1 | F-GAGGCTCTGAGAATCTACTGACT<br>R-CTCGACGGCAACCATACACT | 194 | |
| CD40 | CD40 molecule | NM_001105611.2 | F-TCACCGTTGGACAAGCTGC<br>R-CCGCTTCTTGGTTATGTTCCTG | 184 | |
| IL1B | interleukin 1, beta | NM_174093.1 | F-TTCTCTCCAGCCAACCTTCATT<br>R-ATCTGCAGCTGGATGTTTCCAT | 198 | |
| IL6 | interleukin 6 | NM_173923.2 | F-CGGATGCTTCCAATCTGGGT<br>R-TCTGCGATCTTTTGCTTCAGG | 183 | |
| IL8 (CXCL8) | C-X-C motif chemokine ligand 8 | NM_173925.2 | F-GTGAAGAGAGCTGAGAAGCAAG<br>R-CACCAGACCCACACAGAACAT | 150 | |
| IL10 | interleukin 10 | NM_174088.1 | F-CCTGGAAGAGGTGATGCCAC<br>R-AGGGCAGAAAGCGATGACAG | 125 | |
| LBP | lipopolysaccharide binding protein | NM_001038674.2 | F-ATCGAAGCCTTTGTGCTCCT<br>R-GCTCCACATTGAATCGTCCA | 178 | |
| PTGS2 | prostaglandin-endoperoxide synthase 2 | NM_174445.2 | F-TGAAAACTGTACCACACCCGAA<br>R-ACAGGTTAGAAAAGGCTTCCCA | 242 | |
| TLR4 | toll like receptor 4 | NM_174198.6 | F-CCTTGCGTACAGGTTGTTCC<br>R-TATGGGGATGTTGTCGGGGA | 81 | Heymann et al. 2023, doi:10.1371/journal.pone.0286995 |
| TNF | tumor necrosis factor | NM_173966.3 | F-GGTTCAAACACTCAGGTCCTCT<br>R-GAGGTAAAGCCCGTCAGCAG | 181 | |
| TNFAIP3 | TNF alpha induced protein 3 | NM_001192170.1 | F-GAAATCCCCGTTCAAGGCTG<br>R- CTCTCCGTCCCTGCTCATTG | 166 | |
| **Transcription factors and signal transduction** | | | | | |
| MYC | MYC proto-oncogene, bHLH transcription factor | NM_001046074.2 | F-TACAACATCCGAGCGACACC<br>R-TGCAAGCCCGTATTTCCACT | 167 | |

| | | | | | |
|---|---|---|---|---|---|
| MYD88 | MYD88 innate immune signal transduction adaptor | NM_001014382.2 | F-ATTGAGAAGAGGTGCCGTCG<br>R-ACTTGATGGGGATCAGTCGC | 136 | |
| NFE2L2 | NFE2 like bZIP transcription factor 2 | NM_001011678.2 | F-GCATGATGGACTTGGAGCTG<br>R-GCTCATGCTCCTTCTGTCGT | 144 | |
| NFKB1 | nuclear factor kappa B subunit 1 | NM_001076409.1 | F-CCCCACGTATGGCGGAATTA<br>R-TGCCACAACTTTCAGGGTCA | 110 | Häussler et al. 2023, doi:10.3168/jds.2023-23556 |
| PI3K (PIK3CB) | phosphatidylinositol-4,5-bisphosphate 3-kinase, catalytic subunit beta | NM_001206047.1 | F-AGCGCTGAGCAGTGTATCTT<br>R-GTCCACTGCCCAGATGTCAA | 197 | Häussler et al. 2023, doi:10.3168/jds.2023-23556 |
| PPARA | peroxisome proliferator activated receptor alpha | NM_001034036.1 | F-TGTCGTTTCCACAAGTGCCT<br>R-GGTTGTTGTTGGTCTTCCCG | 244 | |
| SGK1 | serum/glucocorticoid regulated kinase 1 | NM_001102033.1 | F-TCTGGCGATGACGGTGAAAA<br>R-TTCAGGCCCATCCTTCTCTG | 114 | Häussler et al. 2023, doi:10.3168/jds.2023-23556 |
| TICAM1 | TIR domain containing adaptor molecule 1 | NM_001030301.1 | F-TGCTGCTCACCTCCAACTTT<br>R-TAGGGGCAGGAAGGGAATCA | 116 | |
| **Hormone receptors** | | | | | |
| ESR2 | estrogen receptor 2 | NM_174051.3 | F-AGAGTCCTTGGCGTGAAACC<br>R-CCTTCACACGACCAGACTCC | 193 | |
| NR3C1 | nuclear receptor subfamily 3 group C member 1 | NM_001206634.1 | F-AAAGAGCAGTGGAAGGACAGC<br>R-GCGAGACTCCTGTAGTGGC | 189 | Heymann et al. 2023, doi:10.1371/journal.pone.0286995 |
| **Associated with oxidative stress** | | | | | |
| ALOX5 | arachidonate 5-lipoxygenase | NM_001192792.2 | F-CCACAAGGACTTACCCCGAG<br>R-CGGTCTTCCTGCCAGTGATT | 213 | |
| CYBB | cytochrome b-245 beta chain | NM_174035.4 | F-TCGAAAACTTCTTGGGTCAGC<br>R-AGGTGAGGTTCCTGTCCAGT | 173 | |
| EGLN1 | egl-9 family hypoxia inducible factor 1 | NM_001206046.2 | F-GAGAGAGCGCGAGCTAAAGTA<br>R-TAGGTGAAGTGGGGTATTGCTG | 131 | |
| GPX1 | glutathione peroxidase 1 | NM_174076.3 | F-GAGCCCTTCAACCTGTCCTC<br>R-GCGTTTTCCTGATGCCCAAAC | 179 | Bühler et al. 2017, doi:10.1111/jpn.12705 |
| HIF1A | hypoxia inducible factor 1 subunit alpha | NM_174339.3 | F-TCAAGCAGTAGGAATTGGAACA<br>R-ATCCATTGATTGCCCCAGCA | 178 | |
| HMOX1 | heme oxygenase 1 | NM_001014912.1 | F-ATGCCCCAGGATTTGTCAGAG<br>R-GGGGTTCTCCTTGTTGCGTT | 192 | |
| NCF1 | neutrophil cytosolic factor 1 | NM_174119.4 | F-GGAGGGTGAAGCCATTGAGG<br>R-CTGTGAGCATTGCGGATGGA | 189 | Heymann et al. 2023, doi:10.1371/journal.pone.0286995 |
| PARP1 | poly (ADP-ribose) polymerase 1 | NM_174751.2 | F-CGGACAGATGTTTCAGGCAAAG<br>R-TGGGGCTTATCGGGGTACA | 179 | Bühler et al. 2017, doi:10.1111/jpn.12705 |
| RAC1 | Rac family small GTPase 1 | NM_174163.2 | F-ACCCGCAGACAGATGTATTCTT<br>R-AGGTCAAGTTTCGTCCCCAC | 142 | |
| RAC2 | Rac family small GTPase 2 | NM_175792.2 | F-AGGACACCATTGAGAAGCTGAA<br>R-TCAAAACCATCTGGCTGGGAAG | 248 | Heymann et al. 2023, doi:10.1371/journal.pone.0286995 |
| SIRT1 | sirtuin 1 | NM_001192980.3 | F-TGGCACAGATCCTCGAACAA<br>R-ACCCCAGCTCCAGTTAGAAC | 210 | |

| | | | | | |
|---|---|---|---|---|---|
| SIRT2 | sirtuin 2 | NM_001113531.1 | F-AGCGACTCCTGGACGAACTA<br>R-TCCCACCAAACAGATGACCC | 86 | |
| SOD1 | superoxide dismutase 1 | NM_174615.2 | F-GGCAAAGGGAGATACAGTCGT<br>R-CATTGCCCAGGTCTCCAACA | 191 | |
| SOD2 | superoxide dismutase 2, mitochondrial | NM_201527.2 | F-CGTGACTTTGGTTCCTTTGCC<br>R-GCGTCCCTGCTCCTTATTGA | 108 | Bühler et al. 2017, doi:10.1111/jpn.12705 |
| TXN2 | thioredoxin 2 | NM_174208.2 | F-TCGATGACCACACAGACCTC<br>R-GTCCACCACATCCCCATTCTT | 86 | |
| TXNRD1 | thioredoxin reductase 1 | NM_174625.5 | F-CTCCCGGAGCCCTATGACTA<br>R-ACACACGTTCCTCCAAGACC | 161 | |
| XOR (XDH) | xanthine dehydrogenase | NM_173972.2 | F-AAGTCACGGCTCTCAGTGTG<br>R- CCACAGCATCCACCATTCTTG | 192 | Bühler et al. 2017, doi:10.1111/jpn.12705 |
| **Respiratory chain** | | | | | |
| COX4I1 | cytochrome c oxidase subunit 4I1 | NM_001001439.3 | F-TGGCCCATGTCAAGAACCTG<br>R-TGTGCTCCTGTTCATCTCGG | 146 | |
| SDHA | succinate dehydrogenase complex flavoprotein subunit A | NM_174178 | F-TCTACGACACCGTGAAAGGC<br>R-TGCCGTAATTCTCCAGCTCC | 105 | Bühler et al. 2016, doi:10.1016/j.vetimm.2016.07.007 |
| SDHB | succinate dehydrogenase complex iron sulfur subunit B | NM_001040483.1 | F-GATCGACACCAACCTCAGCA<br>R-CGTCCAGTTTCTCACGGTCT | 197 | |
| **Heat-shock response** | | | | | |
| HSP90AA1 | heat shock protein 90 alpha family class A member 1 | NM_001012670.2 | F-ACGATTGGGAGGATCACTTGG<br>R-TCCTCAGAATCCACCACACCT | 226 | |
| HSP90B1 | heat shock protein 90 beta family member 1 | NM_174700.2 | F-GTATGGAGCAGCAAGACCGA<br>R-TCAGTTCCCAATCCCAGACAG | 166 | |
| HSPA1A | heat shock protein family A (Hsp70) member 1A | NM_203322.3 | F-AGGACTTCGACAACAGGCTG<br>R-TGCTGGACGACAAGGTTCTC | 141 | Heymann et al. 2023, doi:10.3390/ani13091499 |
| **Apoptosis** | | | | | |
| BAX | BCL2 associated X, apoptosis regulator | NM_173894.1 | F-GCTGCAGAGGATGATCGCAGCTGTG<br>R-ATCAACTCGGGCACCTTGGTGCA | 174 | Bühler et al. 2016, doi:10.1016/j.vetimm.2016.07.007 |
| BCL2 | BCL2 apoptosis regulator | NM_001166486.1 | F-ACGGAGGCTGGGACGCCTTT<br>R-AGGGTGATGCAAGCGCCCAC | 121 | Bühler et al. 2016, doi:10.1016/j.vetimm.2016.07.007 |
| BCL2L1 | BCL2 like 1 | NM_001077486.2 | F-GGAAAGCGTAGACAAGGAGATG<br>R-CTGCATTGTTCCCGTAGAGTT | 143 | |
| CASP3 | caspase 3 | NM_001077840.1 | F-CAGCGTCGTAGCTGAACGTA<br>R-AGGCCATGCCAGTATTTTCG | 221 | Bühler et al. 2016, doi:10.1016/j.vetimm.2016.07.007 |
| DDIT3 | DNA damage inducible transcript 3 | NM_001078163.1 | F-CCTGAGGAGAGAGTGTTCCAGA<br>R-GATCAGGCTCTGCTTTCAGGT | 70 | Häussler et al. 2023, doi:10.3168/jds.2023-23556 |
| PDCD4 | programmed cell death 4 | NM_001083647.1 | F-GCGGCCTGAGGGAAATACA<br>R-TCCTCAGTCCCAGCATTTTCTT | 180 | Häussler et al. 2023, doi:10.3168/jds.2023-23556 |
| **Tryptophan kynurenine** | | | | | |
| IDO1 | indoleamine 2,3-dioxygenase 1 | NM_001101866.2 | F-GCCCAAAGCAGCATCTTTCA<br>R-AGAACAAACTCACGGACCGA | 179 | |

| | | | | | |
|---|---|---|---|---|---|
| KMO | kynurenine 3-monooxygenase | NM_001243298.1 | F-AGGAATGAATGCGGGCTTTG<br>R-ACCTTGAGTTGACGTGTGCT | 179 | |
| **Mitochondria associated** | | | | | |
| FIS1 | fission, mitochondrial 1 | NM_001034784.2 | F-TAGCGTCTGAGTTGTGAGACC<br>R-ACGGATGTCGTCGTTGTACT | 193 | Häussler et al. 2023, doi:10.3168/jds.2023-23556 |
| MFN2 | mitofusin 2 | NM_001190269.1 | F-CCAGTTGTACCAGCAGACGA<br>R-TCGAGAGAAGAGCAGGGACA | 97 | Häussler et al. 2023, doi:10.3168/jds.2023-23556 |
| **Reference genes** | | | | | |
| ACTB | actin, beta | NM_173979.3 | F-AGATCAAGATCATCGCGCCC<br>R-GCCTAGAAGCATTTGCGGTG | 154 | Bühler et al. 2016, doi:10.1016/j.vetimm.2016.07.007 |
| B2M | beta-2-microglobulin | NM_173893.3 | F-AGCAGCACCATCGAGATTTGA<br>R-TGGACATGTAGCACCCAAGG | 172 | Bühler et al. 2016, doi:10.1016/j.vetimm.2016.07.007 |
| GAPDH | glyceraldehyde-3-phosphate dehydrogenase | NM_001034034.2 | F-ATGCTGGTGCTGAGTATGTGG<br>R-GCAGAAGGTGCAGAGATGATG | 112 | |
| RPLP0 | ribosomal protein lateral stalk subunit P0 | NM_001012682.1 | F-AACTCTGCATTCCCGCTTCC<br>R-GCCTTGACCTTTTCAGCAAGTG | 174 | Bühler et al. 2016, doi:10.1016/j.vetimm.2016.07.007 |
| RPS9 | ribosomal protein S9 | NM_001101152.2 | F-GGCGTCTGTTCGAAGGTAATG<br>R-GGGATGTTCACCACCTGCTT | 229 | Heymann et al. 2023, doi:10.3390/ani13091499 |
| UCHL5 | ubiquitin Cl-terminal hydrolase L5 | NM_174481 | F-CAAAGACAACTTGCTGAGGAACC<br>R-ACTGCTTGTGTTCTGCTAAAGTC | 208 | Bühler et al. 2016, doi:10.1016/j.vetimm.2016.07.007 |
| UXT | ubiquitously expressed prefoldin like chaperone | NM_001037471.2 | F-CGCTACGAGGCTTTCATCTCT<br>R-CGAGTGGTTAGCTTCCTGGAGT | 141 | Heymann et al. 2021, doi:10.1371/journal.pone.0246679 |
| YWHAZ | tyrosine 3-monooxygenase/tryptophan 5-monooxygenase activation protein zeta | NM_174814.2 | F-CGGACACAGAACATCCAGTC<br>R-TCCAAGATGACCTACGGGCT | 198 | Bühler et al. 2016, doi:10.1016/j.vetimm.2016.07.007 |
| **Not evaluable** | | | | | |
| ACACA | acetyl-CoA carboxylase alpha | NM_174224.2 | F-ATCCGACGCCTTACTTTCCT<br>R-TTCTCATCCGGTTCAGCTCT | 193 | |
| CPT1B | carnitine palmitoyltransferase 1B | NM_001034349.2 | F-AGCTCAGAGACAAATGCCCA<br>R-ATCAGCCTTGGGAACTTGGAA | 90 | |
| CPT2 | carnitine palmitoyltransferase 2 | NM_001045889.2 | F-CGGCCTGATCTCCATACACC<br>R-CTGGTAGTGCATAGTGGGCA | 205 | Ghaffari et al. 2021, doi:10.3168/jds.2021-20237 |
| CYBA | cytochrome b-245 alpha chain | NM_174034.2 | F-TCCATAGCAGCAGGCGTATT<br>R-CTTCTGTCCACACCTCTCCAT | 90 | |
| GLUT1 (SLC2A1) | solute carrier family 2 member 1 | NM_174602.2 | F-CCTTCACTGTCGTGTCGCTAT<br>R-GCCACAATGCTCAGGTAGGA | 157 | |
| MPO | myeloperoxidase | NM_001113298.2 | F-GAACCGCCAGAACCAAATCG<br>R-AGTTTCATCAGCCTCCTCGC | 228 | |
| NOS2 | nitric oxide synthase 2 | NM_001076799.1 | F-GCCTCTGGACCTCAACAAAGC<br>R-CATCCACCACTCGCTCAAGG | 233 | |
| OPA1 | mitochondrial dynamin like GTPase | NM_001192961.1 | F-CCTTGCAAAATTGGCACCTGA<br>R-CCAGGTGAACCTGTGGTGAA | 84 | Häussler et al. 2023, doi:10.3168/jds.2023-23556 |

| | | | | | |
|---|---|---|---|---|---|
| PTGS1 | prostaglandin-endoperoxide synthase 1 | NM_001105323.1 | F-TCTGTCTCCAGCACGCAAC<br>R-TGCAGTCACATTGGTAGCGG | 211 | |
| RELA | v-re reticuloendotheliosis viral oncogene homolog A (Avian) / NF-kappaB transcription factor p65 subunit | NM_001080242.2 | F-AACAACCCCTTCCAAGTTCCC<br>R-CCCAGAGTTCCGATTCACCC | 201 | Bühler et al. 2016, doi:10.1016/j.vetimm.2016.07.007 |
| SIRT3 | sirtuin 3 | NM_001206669.1 | F-TGGCGTTGTTTCCTCGTTCA<br>R-CACTTGAGGCACCAGCAAAA | 132 | Häussler et al. 2023, doi:10.3168/jds.2023-23556 |
| SLC27A2 | solute carrier family 27 member 2 | NM_001192863.1 | F-ATGGCGTGCCTCAACTACAA<br>R-GAGTTGACCCCATCCGTGTT | 188 | Ghaffari et al. 2021, doi:10.3168/jds.2021-20237 |
| UCP2 | uncoupling protein 2 | NM_001033611.2 | F-CCTCTACGACTCCGTCAAGC<br>R-GAGTCCCCTAAACCCCTCCT | 232 | Häussler et al. 2023, doi:10.3168/jds.2023-23556 |

**Supplementary Table 2:** Effects of dietary L-carnitine supplementation from 11 days before to 14 days after intravenous LPS injection on calibrated normalized relative quantities of gene expression in peripheral blood mononuclear cells from dairy cows. Measured with real-time qPCR. Data are shown as least square means. See **Supplementary Table 1** for abbreviations.

| | **Time points[2]** | | | ***p*-value** | | | |
|---|---|---|---|---|---|---|---|
| **Gene CNRQ[1]** | **-11 d** | **24 h** | **14 d** | **Group** | **Time** | **Group x Time** | **PSE[3]** |
| **Carnitine associated** | | | | | | | |
| CPT1A | 2.881 | 6.503 | 2.223 | 0.212 | **0.001** | 0.739 | 1.163 |
| CROT | 0.892 | 0.752 | 1.335 | 0.833 | **0.003** | 0.885 | 0.278 |
| SLC22A5 | 2.021 | 1.836 | 1.805 | 0.880 | 0.050 | 0.932 | 0.166 |
| SLC25A20 | 3.132 | 3.898 | 2.722 | 0.281 | **<0.001** | **0.036** | 0.421 |
| TMLHE | 1.234 | 1.312 | 1.225 | 0.981 | **0.047** | 0.896 | 0.034 |
| **Energy metabolism** | | | | | | | |
| ACOX1 | 1.223 | 1.494 | 1.169 | 0.580 | **0.006** | 0.810 | 0.147 |
| G6PD | 1.326 | 1.315 | 1.343 | 0.373 | 0.767 | 0.308 | 0.081 |
| LDHA | 0.535 | 0.754 | 0.774 | 0.618 | **0.005** | 0.156 | 0.108 |
| LDHB | 0.801 | 0.816 | 0.767 | 0.725 | 0.177 | 0.196 | 0.028 |
| PDHA1 | 2.113 | 1.878 | 1.816 | 0.995 | **0.026** | 0.085 | 0.106 |
| PDK1 | 3.708 | 2.782 | 3.291 | 0.888 | **<0.001** | 0.321 | 0.178 |
| PDP1 | 1.509 | 1.212 | 1.210 | 0.116 | **<0.001** | 0.063 | 0.073 |
| PDPR | 2.426 | 2.158 | 2.225 | 0.925 | **0.005** | 0.283 | 0.064 |
| PKM | 0.671 | 0.909 | 0.724 | 0.927 | **<0.001** | 0.481 | 0.038 |
| PRKAA1 | 1.091 | 0.929 | 0.999 | 0.730 | **0.006** | 0.482 | 0.075 |
| PRKAB1 | 1.921 | 1.812 | 1.586 | 0.955 | **0.029** | 0.942 | 0.277 |
| PRKAG1 | 2.137 | 2.027 | 1.970 | 0.982 | **0.022** | 0.468 | 0.055 |
| TKT | 0.807 | 1.130 | 0.850 | 0.426 | **<0.001** | 0.968 | 0.065 |
| **Transporters and receptors** | | | | | | | |
| FFAR2 | 0.107 | 0.18 | 0.12 | 0.196 | **0.025** | 0.498 | 0.030 |
| GLUT3 | 0.249 | 0.270 | 0.251 | 0.510 | 0.207 | 0.346 | 0.072 |
| **Immune response** | | | | | | | |
| CAT | 2.829 | 2.690 | 2.612 | 0.448 | 0.070 | 0.788 | 0.075 |
| CD14 | 1.072 | 1.736 | 1.139 | 0.430 | **0.002** | 0.175 | 0.218 |
| CD40 | 2.106 | 1.965 | 1.916 | 0.677 | **0.016** | 0.178 | 0.149 |
| IL1B | 0.082 | 0.037 | 0.075 | 0.412 | **<0.001** | 0.405 | 0.012 |
| IL6 | 1.256 | 2.127 | 2.350 | 0.756 | **<0.001** | 0.880 | 0.523 |
| IL8 | 0.676 | 0.967 | 0.878 | 0.780 | **0.006** | 0.619 | 0.122 |
| IL10 | 0.420 | 0.436 | 0.259 | 0.807 | **0.001** | 0.254 | 0.079 |
| LBP | 0.264 | 0.310 | 0.314 | 0.178 | 0.855 | 0.178 | 0.172 |
| PTGS2 | 3.906 | 3.600 | 3.346 | 0.866 | 0.387 | 0.371 | 0.367 |
| TLR4 | 0.310 | 0.205 | 0.350 | 0.657 | **<0.001** | 0.557 | 0.016 |

| | | | | | | | |
|---|---|---|---|---|---|---|---|
| TNF | 3.983 | 4.399 | 4.357 | 0.871 | 0.131 | 0.601 | 0.217 |
| TNFAIP3 | 0.519 | 0.334 | 0.455 | 0.725 | **<0.001** | 0.701 | 0.042 |
| **Transcription factors and signal transduction** | | | | | | | |
| MYC | 1.098 | 1.180 | 1.260 | 0.948 | 0.200 | 0.202 | 0.083 |
| MYD88 | 0.371 | 0.429 | 0.374 | 0.445 | **0.001** | 0.903 | 0.026 |
| NFE2L2 | 1.386 | 1.192 | 1.526 | 0.740 | **0.001** | 0.934 | 0.066 |
| NFKB1 | 0.854 | 0.915 | 0.855 | 0.859 | 0.166 | 0.849 | 0.025 |
| PI3K | 1.603 | 1.953 | 1.757 | 0.960 | **<0.001** | 0.949 | 0.075 |
| PPARA | 3.218 | 2.411 | 3.318 | 0.849 | **<0.001** | 0.305 | 0.114 |
| SGK1 | 0.778 | 1.355 | 0.492 | 0.637 | **<0.001** | 0.913 | 0.149 |
| TICAM1 | 4.161 | 4.244 | 4.087 | 0.879 | 0.633 | 0.293 | 0.158 |
| **Hormone receptors** | | | | | | | |
| ESR2 | 1.984 | 1.943 | 1.773 | 0.658 | 0.715 | 0.518 | 0.564 |
| NR3C1 | 1.281 | 1.104 | 1.212 | 0.916 | **<0.001** | 0.271 | 0.045 |
| **Associated with oxidative stress** | | | | | | | |
| ALOX5 | 0.013 | 0.036 | 0.011 | 0.197 | **0.037** | 0.400 | 0.026 |
| CYBB | 1.356 | 1.311 | 1.346 | 0.338 | 0.624 | 0.805 | 0.045 |
| EGLN1 | 1.984 | 1.951 | 1.872 | 0.975 | 0.366 | 0.547 | 0.067 |
| GPX1 | 3.318 | 5.265 | 3.298 | 0.468 | **<0.001** | 0.295 | 0.472 |
| HIF1A | 2.084 | 1.704 | 1.903 | 0.815 | **0.001** | 0.351 | 0.187 |
| HMOX1 | 1.031 | 2.092 | 1.045 | 0.555 | **<0.001** | 0.611 | 0.168 |
| NCF1 | 0.201 | 0.175 | 0.178 | 0.855 | **0.036** | 0.862 | 0.010 |
| PARP1 | 2.241 | 1.990 | 1.943 | 0.643 | **<0.001** | 0.151 | 0.147 |
| RAC1 | 1.039 | 1.056 | 1.055 | 0.474 | 0.777 | 0.428 | 0.026 |
| RAC2 | 2.979 | 2.987 | 2.746 | 0.743 | 0.359 | 0.509 | 0.174 |
| SIRT1 | 1.125 | 0.841 | 0.909 | 0.113 | **<0.001** | 0.108 | 0.065 |
| SIRT2 | 7.336 | 8.003 | 6.476 | 0.933 | **0.043** | 0.911 | 1.631 |
| SOD1 | 1.765 | 1.636 | 1.754 | 0.652 | **0.018** | 0.668 | 0.044 |
| SOD2 | 0.053 | 0.058 | 0.054 | 0.854 | 0.547 | 0.348 | 0.007 |
| TXN2 | 3.494 | 3.565 | 3.455 | 0.757 | 0.630 | 0.353 | 0.133 |
| TXNRD1 | 1.384 | 1.242 | 1.192 | 0.292 | **<0.001** | 0.552 | 0.031 |
| XOR | 1.820 | 2.182 | 1.915 | 0.261 | **0.007** | 0.620 | 0.084 |
| **Respiratory chain** | | | | | | | |
| COX4I1 | 1.884 | 1.694 | 1.798 | 0.581 | **<0.001** | **0.017** | 0.037 |
| SDHA | 1.852 | 1.791 | 1.648 | 0.431 | **0.001** | 0.391 | 0.046 |
| SDHB | 2.812 | 2.651 | 2.629 | 0.900 | 0.060 | 0.589 | 0.074 |
| **Heat-shock response** | | | | | | | |
| HSP90AA1 | 0.556 | 0.510 | 0.512 | 0.753 | **0.001** | 0.172 | 0.027 |
| HSP90B1 | 1.598 | 1.263 | 1.411 | 0.639 | **<0.001** | 0.268 | 0.053 |
| HSPA1A | 1.868 | 2.108 | 2.147 | 0.354 | **0.032** | 0.553 | 0.111 |
| **Apoptosis** | | | | | | | |

| | | | | | | | |
|---|---|---|---|---|---|---|---|
| BAX | 3.847 | 3.725 | 3.689 | 0.341 | 0.842 | 0.266 | 0.254 |
| BCL2 | 2.586 | 2.495 | 2.255 | 0.381 | **0.007** | 0.307 | 0.086 |
| BCL2L1 | 1.201 | 1.719 | 1.636 | 0.505 | **<0.001** | 0.811 | 0.254 |
| CASP3 | 0.828 | 1.336 | 1.028 | 0.867 | **0.001** | 0.172 | 0.166 |
| DDIT3 | 1.403 | 1.329 | 1.414 | 0.966 | 0.360 | 0.976 | 0.064 |
| PDCD4 | 2.112 | 1.961 | 2.018 | 0.437 | 0.110 | 0.881 | 0.063 |
| **Tryptophan kynurenine** | | | | | | | |
| IDO1 | 1.938 | 0.671 | 1.873 | 0.928 | **<0.001** | 0.953 | 0.280 |
| KMO | 0.318 | 0.539 | 0.300 | 0.065 | **0.039** | 0.253 | 0.103 |
| **Mitochondria associated** | | | | | | | |
| FIS1 | 2.058 | 2.110 | 1.933 | 0.358 | **0.011** | 0.348 | 0.110 |
| MFN2 | 1.589 | 0.994 | 2.022 | 0.610 | **<0.001** | 0.787 | 0.376 |

[1] calibrated normalized relative quantities, [2] days / hours relative to LPS injection, [3] pooled standard error

**Supplementary Table 3:** Effects of *ex vivo* stimulus (unstimulated, concanavalin A = ConA or LPS) and dietary L-carnitine supplementation from 11 days before to 14 days after intravenous LPS injection on key parameters of mitochondrial functionality of peripheral blood mononuclear cells from dairy cows. Measured with Seahorse analyzer and normalized to DNA content. Data are shown as least square means.

| **Time[1] / Stimulus[2]** | **Spare respiratory capacity [OCR[3] normalized]** | **Spare respiratory capacity [%]** | **Coupling efficiency [%]** | **Bioenergetic Health Index** |
|---|---|---|---|---|
| -11 d / unstimulated | 3.257 | 176.2 | 79.81 | 0.873 |
| -11 d / ConA | 2.006 | 146.7 | 79.96 | 0.700 |
| -11 d / LPS | 1.538 | 140.2 | 81.21 | 0.732 |
| 24 h / unstimulated | 2.319 | 158.4 | 79.55 | 0.765 |
| 24 h / ConA | 1.538 | 140.4 | 77,87 | 0.784 |
| 24 h / LPS | 0.977 | 128.8 | 78.81 | 0.694 |
| 14 d / unstimulated | 3.269 | 173.6 | 73.30 | 0.658 |
| 14 d / ConA | 2.143 | 141.1 | 70.05 | 0.493 |
| 14 d / LPS | 0.738 | 115.5 | 74.97 | 0.438 |
| *p*-value | | | | |
| Group | 0.320 | 0.684 | 0.574 | 0.553 |
| Time | 0.068 | 0.144 | **<0.001** | **0.011** |
| Stimulus | **<0.001** | **<0.001** | 0.298 | 0.095 |
| Group x Time | 0.480 | 0.953 | 0.690 | 0.170 |
| Group x Stimulus | 0.737 | 0.817 | 0.782 | 0.433 |
| Time x Stimulus | 0.629 | 0.586 | 0.807 | 0.658 |
| Group x Time x Stimulus | 0.977 | 0.902 | 0.683 | 0.588 |
| PSE[4] | 0.163 | 4.473 | 0.779 | 0.038 |

[1] days / hours relative to LPS injection, [2] *ex vivo* stimulus (unstimulated, concanavalin A = ConA or LPS), [3] oxygen consumption rate, [4] pooled standard error
